# RNF126‐Mediated MRE11 Ubiquitination Activates the DNA Damage Response and Confers Resistance of Triple‐Negative Breast Cancer to Radiotherapy

**DOI:** 10.1002/advs.202203884

**Published:** 2022-12-23

**Authors:** Wenjing Liu, Min Zheng, Rou Zhang, Qiuyun Jiang, Guangshi Du, Yingying Wu, Chuanyu Yang, Fubing Li, Wei Li, Luzhen Wang, Jiao Wu, Lei Shi, Wenhui Li, Kai Zhang, Zhongmei Zhou, Rong Liu, Yingzheng Gao, Xinwei Huang, Songqing Fan, Xu Zhi, Dewei Jiang, Ceshi Chen

**Affiliations:** ^1^ Key Laboratory of Animal Models and Human Disease Mechanisms of the Chinese Academy of Sciences and Yunnan Province Kunming Institute of Zoology Chinese Academy of Sciences Kunming 650201 China; ^2^ Kunming College of Life Sciences University of the Chinese Academy of Sciences Kunming 650204 China; ^3^ The Third Affiliated Hospital Kunming Medical University Kunming 650118 China; ^4^ Department of the Pathology First Affiliated Hospital of Kunming Medical University Kunming 650032 China; ^5^ Academy of Biomedical Engineering Kunming Medical University Kunming 650500 China; ^6^ School of Life Science University of Science & Technology of China Hefei 230027 China; ^7^ Department of Biochemistry and Molecular Biology Tianjin Medical University Tianjin 300070 China; ^8^ Translational Cancer Research Center Peking University First Hospital Beijing 100034 China; ^9^ Department of the Central Laboratory Second Affiliated Hospital of Kunming Medical University Kunming 650032 China; ^10^ Department of Pathology the Second Xiangya Hospital Central South University Changsha 410000 China; ^11^ Center for Reproductive Medicine Department of Obstetrics and Gynecology Peking University Third Hospital Beijing 100191 China

**Keywords:** ataxia telangiectasia mutated and rad‐3 related protein kinase (ATR), dihydroartemisinin, homologous recombination repair, human epidermal growth factor receptor 2 (HER2), Meiotic recombination 11 homolog 1 (MRE11), Nuclear Factor Kappa‐Β (NF‐*κ*B), Ring finger protein 126 (RNF126)

## Abstract

Triple‐negative breast cancer (TNBC) has higher molecular heterogeneity and metastatic potential and the poorest prognosis. Because of limited therapeutics against TNBC, irradiation (IR) therapy is still a common treatment option for patients with lymph nodes or brain metastasis. Thus, it is urgent to develop strategies to enhance the sensitivity of TNBC tumors to low‐dose IR. Here, the authors report that E3 ubiquitin ligase Ring finger protein 126 (RNF126) is important for IR‐induced ATR‐CHK1 pathway activation to enhance DNA damage repair (DDR). Mechanistically, RNF126 physically associates with the MRE11‐RAD50‐NBS1 (MRN) complex and ubiquitinates MRE11 at K339 and K480 to increase its DNA exonuclease activity, subsequent RPA binding, and ATR phosphorylation, promoting sustained DDR in a homologous recombination repair‐prone manner. Accordingly, depletion of RNF126 leads to increased genomic instability and radiation sensitivity in both TNBC cells and mice. Furthermore, it is found that RNF126 expression is induced by IR activating the HER2‐AKT‐NF‐*κ*B pathway and targeting RNF126 expression with dihydroartemisinin significantly improves the sensitivity of TNBC tumors in the brain to IR treatment in vivo. Together, these results reveal that RNF126‐mediated MRE11 ubiquitination is a critical regulator of the DDR, which provides a promising target for improving the sensitivity of TNBC to radiotherapy.

## Graphical abstract

RNF126 is first reported to mediate the non‐degradative ubiquitination of MRE11 to facilitate its exonuclease activity and promote homologous recombination repair, which is induced by the IR‐activating HER2‐AKT‐NF‐*κ*B pathway in TNBC cells. Dihydroartemisinin, a potent antimalarial agent, is proven to inhibit RNF126 expression and enhance radiotherapy sensitization on mouse model with a TNBC tumor in the brain, exhibiting high potential in clinical application.

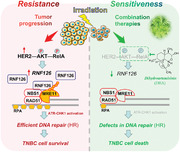

## 1 Introduction

Breast cancer has become the leading cause of cancer incidence worldwide, and the incidence and mortality rate of breast cancer were the highest in most countries among women in 2020.^[^ ^1^ ^]^ Triple‐negative breast cancer (TNBC), lacking the expression of estrogen receptor (ERα), progesterone receptor (PR), and human epidermal growth factor receptor 2 (HER2), accounts for 15%‐20% of breast cancer incidence. TNBC has a poorer prognosis than other subtypes of breast cancer due to higher proliferation, higher rates of recurrence and metastasis, and the absence of effective targeted drugs.^[^ ^2^ ^]^ Irradiation (IR) therapy, which is well established to improve locoregional control in breast cancer patients with a positive impact on high‐risk patients for long‐term survival, is one of the primary therapeutic strategies for TNBC.^[^ ^3^ ^]^ However, high‐energy radiation simultaneously and inevitably causes damage to the patient's normal tissues. Therefore, therapeutic strategies achieving optimal outcomes of radiotherapy at the lowest possible cost by improving sensitivity, reducing toxicity, and increasing convenience have attracted increasing attention in recent years.

Both normal and cancer cells possess an intricate signaling network to overcome the threat of genomic instability, namely, the DNA damage response (DDR) system.^[^ ^4^, ^5^ ^]^ In most cancers, one or more DDR pathways or capabilities are lost, resulting in greater dependence on the remaining pathways.^[^ ^6^ ^]^ This provides the opportunity to increase radiation sensitivity by targeting the DDR. Irradiation causes cell death primarily by inducing DNA damage, particularly DNA double‐strand breaks (DSBs). There are two different pathways to repair DSBs in eukaryotic cells.^[^ ^7^ ^]^ Homologous recombination (HR) repair utilizes homologous DNA as a template to complete the repair of damaged or missing DNA sequences in a high‐fidelity manner.^[^ ^8^ ^]^ Nonhomologous end joining (NHEJ) repair directly connects the ends of DSBs without the need for templates and is more extensively used but more error‐prone.^[^ ^9^ ^]^ The balance between these different pathways is essential for correct DSB repair.^[^ ^10^ ^]^ However, the mechanism by which HR and NHEJ are chosen is not completely clear.

The MRN complex is of great importance to maintain genomic stability and support normal cell function.^[^ ^11^ ^]^ The MRN complex consists of MRE11, RAD50, and NBS1, in which dimeric MRE11 forms the core of the complex. MRE11 possesses dsDNA 3"‐5" exonuclease activity and ssDNA endonuclease activity to conduct DSB end resection and 3' overhang generation.^[^ ^11^, ^12^ ^]^ During the DNA damage response and repair, the MRN complex is recruited to DSB sites by activating ATM signaling^[^ ^13^, ^14^ ^]^ and is also necessary for the recruitment and activation of ATR in response to IR‐induced DNA damage.^[^ ^15^, ^16^ ^]^

RNF126 belongs to the RING‐type family of E3 ubiquitin ligases and plays a key role in several biological processes, including DNA damage response and repair, cancer development, cytoplasmic protein quality control, and intracellular protein sorting.^[^ ^17^ ^]^ RNF126 has been implicated in DDR. RNF126 directly interacts with the transcription factor E2F1 to promote the transcriptional activation of *BRCA1*, and inhibition of RNF126 increases the sensitivity of cells to PARP (poly ADP‐ribose polymerase) inhibitors.^[^ ^18^ ^]^ Moreover, RNF126 preferentially ubiquitinates Ku80 protein to release Ku70/80 from damaged DNA double strands and causes their degradation, which facilitates the completion of NHEJ repair.^[^ ^19^ ^]^ In contrast, Lee et al. reported RNF126 as a negative regulator of the DNA damage response.^[^ ^20^ ^]^ During the formation of DNA damage induced by IR, RNF126 functions between RNF8 and RNF168 inhibit RNF168‐mediated monoubiquitination of K13/15 in H2A and consequently impair NHEJ repair.^[^ ^20^ ^]^ In the case of UV damage, RNF126 was reported to be recruited to DSBs in an RNF8‐dependent manner and ubiquitinate RNF168 to inhibit RNF168‐mediated *γ*H2AX ubiquitination and the recruitment of downstream DDR factors.^[^ ^21^ ^]^ However, the roles of RNF126 in the DDR remain controversial, and its functions in vivo remain largely unknown. If RNF126 is involved in both HR and NHEJ, how does RNF126 balance the two repair modes when DSBs occur? How is RNF126 regulated in response to IR? Can we pharmacologically target RNF126 to increase IR sensitivity in TNBC?

In this study, we constructed a *Rnf126* knockout (KO) mouse model and demonstrated that Rnf126 promotes mouse survival upon IR treatment. We further found that RNF126 is one of the regulators of IR‐induced DDR activation by ubiquitinating and activating MRE11 to promote RPA‐ATR‐CHK1 signaling cascades. Remarkably, we showed that dihydroartemisinin (DHA) inhibits IR‐induced RNF126 expression by disrupting HER2‐AKT‐NF‐*κ*B signaling and improves the efficacy of IR treatment against TNBC tumors in the brain. Clinically, high expression of *RNF126* is specifically associated with poor prognosis in TNBC patients. Our findings provide promising targets for improving the sensitivity of TNBC to IR therapy.

## 2 Results

### 2.1 Rnf126 Knockout Increased the Sensitivity of Mice to Irradiation, and TNBC Patients with a High RNF126 Expression Level Had a Poorer Prognosis

**Figure 1:**
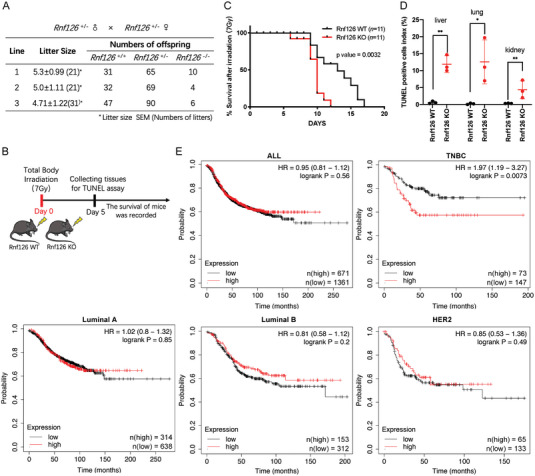
Rnf126 is important for radiation tolerance in mice and TNBC patients with high *RNF126* expression had poorer prognoses. A) *Rnf126* whole‐body knockout (KO) caused partially embryonic lethality. Statistical table of *Rnf126* whole‐body KO mice. B) Schematic diagram for the constructions of whole‐body irradiation of Rnf126 KO mice. C) The survival time of *Rnf126* knockout mice became shorter after irradiation. Kaplan‐Meier survival curves of irradiated *Rnf126* WT versus *Rnf126* KO male C57Bl/6 mice (*n* = 11 per genotype). Statistical analysis was performed using the log‐rank test. D) The apoptotic cells in the tissues of *Rnf126* KO mice increased significantly after irradiation. The quantification of TUNEL staining of livers, lungs, and kidneys isolated from three paired male mice receiving the indicated treatment. Data are mean ± SD. Statistical analysis was performed using a two‐tailed unpaired *t*‐test (*n* = 3 per genotype). * *p* < 0.05 and ** *p* < 0.01; n.s, not significant. E) TNBC patients with high expression of *RNF126* (upper tertile) have a poorer prognosis. Overall survival of different subtypes (PM50) of breast cancer according to high or low *RNF126* mRNA expression levels. The analysis was performed based on the TCGA dataset.

To evaluate the role of RNF126 in the DDR in vivo, we generated a *Rnf126* whole‐body KO mouse model (Figure S1A,B, Supporting Information). We found that *Rnf126* KO had no effect on the body weight of young mice (aged 2 months) but caused partial embryonic lethality (**Figure** **1**A and Figure S1C, Supporting Information). Eleven pairs of two‐month‐old *Rnf126* wild‐type (WT) and whole‐body KO mice were treated with IR treatment at 7 Gy, a lethal dose that kills all WT mice by approximately 3 weeks (Figure 1B). We found that the survival time of the *Rnf126* KO group was significantly shorter than that of the *Rnf126* WT group (Figure 1C). On the seventh day after IR exposure, several mouse tissues were collected, and the TUNEL assay revealed that *Rnf126* KO mice showed a markedly higher proportion of apoptosis (Figure 1D and Figure S1D, Supporting Information).

Our previous study suggests that RNF126 protein expression is upregulated in invasive breast cancer.^[^ ^22^ ^]^ In addition, analyses using TCGA data showed that the expression level of *RNF126* mRNA was not different among breast cancer subtypes (Figure S1E, Supporting Information); however, patients with high *RNF126* expression displayed poorer prognosis than those with low *RNF126* expression only in TNBC (Figure 1E). These results suggest that RNF126 may play an important role in radiation tolerance and that RNF126 is a prognostic marker for TNBC patients.

### 2.2 RNF126 Promotes IR‐Induced ATR‐CHK1 Activation Depending on its E3 Ligase Activity

**Figure 2:**
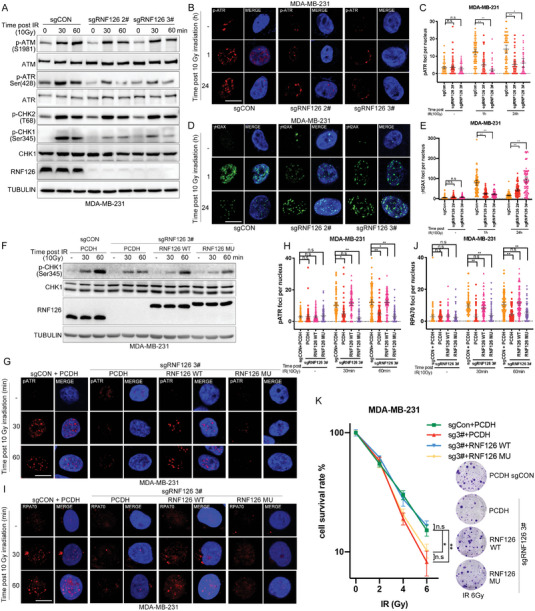
RNF126 activates IR‐induced ATR‐CHK1 signaling depending on its E3 ligase activity. A) *RNF126* KO decreased the ATR‐CHK1 activation post‐irradiation in MDA‐MB‐231 cells. *RNF126* KO MDA‐MB‐231 cells treated with IR (10 Gy) and cell lysates were harvested at 0, 30, and 60 min for Western blotting analysis. B,C) *RNF126* knockout decreased IR‐induced p‐ATR foci formation in MDA‐MB‐231 cells. Immunostaining analysis of p‐ATR foci formation after irradiation (10 Gy) at 0, 1, and 24 h in *RNF126* KO MDA‐MB‐231 cells followed by quantification. Data are mean ± 95% confidence interval (CI) Statistical analysis was performed using two‐tailed unpaired *t‐*s. Each point represents one cell and 100 cells quantified in each group were obtained from one experiment. Data are representative of three independent experiments. * *p* < 0.05 and ** *p* < 0.01; n.s, not significant. Scale bars, 10 µm. D,E) RNF126 knockout increased IR‐induced *γ*H2AX accumulation in MDA‐MB‐231 cells. Immunostaining analysis of *γ*H2AX foci formation after irradiation (10 Gy) at 0, 1, and 24 h in RNF126 stable KO MDA‐MB‐231 cells followed by quantification. Data are mean ± 95% confidence interval (CI) Statistical analysis was performed using two‐tailed unpaired *t*‐tests. Each point represents a cell. Each point represents a cell; 100 cells quantified in each group were obtained from one experiment. Data are representative of three independent experiments. * *p* < 0.05 and ** *p* < 0.01; n.s, not significant. Scale bars, 10 µm. F) RNF126 KO‐impaired CHK1 (p‐Ser345) signals reversed by re‐expressing WT RNF126, but not catalytic inactive RNF126 mutant (C229A/C232A), in MDA‐MB‐231 cells. RNF126 stable KO MDA‐MB‐231 cells with re‐expressing wild type or catalytic inactive RNF126 were treated with IR (10 Gy) and cell lysates were harvested for Western blotting analysis at 0, 30, and 60 min points. PCDH was used as a negative control for RNF126 and sgCON was used as a negative control for sgRNF126. G‐J) RNF126 KO‐decreased ATR (p‐Ser428) and RPA70 foci formation reversed by re‐expressing WT RNF126 but not RNF126 mutant (C229A/C232A). Immunostaining analysis of p‐ATR (G, H) and RPA70 I,J) foci formation after irradiation (10 Gy) at 0, 30, and 60 min points in indicated MDA‐MB‐231 cells followed by quantification. Data are mean ± 95% CI. Statistical analysis was performed using two‐tailed unpaired *t*‐tests. Each point represents a cell. 100 cells quantified in each group were obtained from one experiment. Data are representative of three independent experiments. * *p* < 0.05 and ** *p* < 0.01; n.s, not significant. Scale bars, 10 µm. K) WT RNF126 over‐expression restored IR resistance in RNF126 stable KO MDA‐MB‐231 cells. Clonogenic survival in response to IR of the indicated MDA‐MB‐231 cell lines. Data are mean ± SD. Statistical analysis was performed using two‐tailed unpaired *t*‐tests. Data are representative of three independent experiments. * *p* < 0.05 and ** *p* < 0.01; n.s, not significant.

Due to the defects in DDR in most TNBC, there is greater reliance on ATR pathway signaling following DNA damage, including IR‐induced DSBs.^[^ ^23^, ^24^, ^25^ ^]^ Thus, we sought to test whether RNF126 regulates the ATR signaling pathway. We found that *RNF126* KO substantially decreased ATR (p‐Ser428) and CHK1 (p‐Ser345) activation post‐irradiation but had little effect on ATM‐CHK2 activation in MDA‐MB‐231 and HCC1806 cells (**Figure** **2**A and Figure S2A, Supporting Information). Notably, immunofluorescence staining revealed that *RNF126* KO decreased the formation of ATR (p‐Ser428) foci after 1 or 24 h of recovery in both MDA‐MB‐231 (Figure 2B,C) and HCC1806 (Figure S2B,C, Supporting Information) cells. Consistently, we observed fewer *γ*H2AX foci in *RNF126* KO cells at 1 h post‐irradiation, whereas a greater level of *γ*H2AX signal was observed at 24 h post‐irradiation, indicating a weakening initiation of DDR and retained DNA damage resulting from impaired repair efficiency (Figure 2D,E and Figure S2D,E, Supporting Information). In agreement with this, at 48 h post‐irradiation, the percentage of apoptotic cells was higher in RNF126 KO cells than that in control cells (Figure S2F,G, Supporting Information). These results suggest that depletion of RNF126 results in attenuation in IR‐induced ATR‐CHK1 activation and accumulation of DNA damage.

Then, we wondered whether E3 ligase activity is important for RNF126 to activate ATR‐CHK1 signaling by IR. We found that RNF126 KO‐impaired CHK1 (p‐Ser345) signals could be reversed by re‐expressing WT RNF126 but not the catalytically inactive RNF126 mutant (C229A/C232A) in MDA‐MB‐231 cells (Figure 2F). Consistently, immunofluorescence staining indicated that RNF126 KO‐decreased ATR (p‐Ser428) and RPA70 foci formation could be reversed by re‐expressing WT RNF126 but not the RNF126 mutant (C229A/C232A) (Figure 2G–J). Moreover, a colony formation assay in MDA‐MB‐231 cells showed that depletion of RNF126 significantly increased its irradiation sensitivity and re‐expressed WT RNF126, but not its catalytically inactive mutant, eliminating this effect (Figure 2K). These results indicate that RNF126 activates ATR‐CHK1 signaling upon IR in an E3 ligase activity‐dependent manner.

### 2.3 RNF126 is Physically Associated with the MRN Complex and ATR

**Figure 3:**
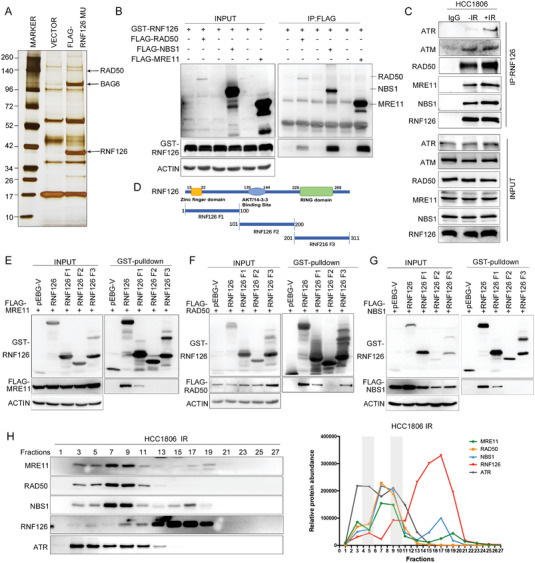
RNF126 is physically associated with MRN and ATR. A) Immunopurification and mass spectrometric analysis of RNF126‐containing protein complexes. Cellular extracts from HCC1806 cells stably expressing FLAG‐RNF126 C292/232A were immunopurified with anti‐FLAG affinity beads and eluted with FLAG peptide. The eluates were resolved on SDS/PAGE and silver stained, followed by mass spectrometric analysis. B) GST‐RNF126 was co‐immunoprecipitated by each FLAG‐tagged MRN protein. GST fused RNF126 and FLAG‐RAD50, FLAG‐NBS1, or FLAG‐MRE11 were expressed in HEK293T cells. GST‐pulldown was used to detect the association between RNF126 and the MRN complex. C) Endogenous RNF126 interacted with MRN, ATM, and ATR. HCC1806 cells were treated with or without IR (10 Gy), and 1 h after IR, cellular extracts were harvested for coimmunoprecipitation followed by Western blotting analysis. D) Schematic of RNF126 fragments. E–G) N‐terminus of RNF126 interacted with MRE11 and NBS1. Map of E) MRE11, F) RAD50, or G) NBS1 interaction domains of RNF126. Different GST‐fused RNF126 fragments and FLAG‐MRE11, FLAG‐RAD50, or FLAG‐NBS1 were expressed in HEK293T cells. Then GST‐pulldown was used to detect the association between RNF126 fragments and the MRN complex. H) Native RNF126 was co‐eluted with MRN complex and ATR. FPLC analysis of the native protein complex. Cellular extracts from HCC1806 cells after 1 h post‐IR (10 Gy) were fractionated on Superose 6 size‐exclusion columns with a high‐salt buffer. Western blotting analysis of the chromatographic fractions with antibodies against the indicated proteins. Equal volumes from each fraction were analyzed and the boxed area indicates fractions in which endogenous RNF126 was co‐eluted with the MRN complex. The amounts of the indicated proteins were quantified using ImageJ.

To further understand the mechanism by which RNF126 confers IR‐induced ATR‐CHK1 activation, we performed affinity purification and mass spectrometry in HCC1806 cells with stable overexpression of the catalytically inactive RNF126 mutant to identify its interacting proteins. As a result, RNF126 may interact with RAD50, a component of the MRN complex (**Figure** **3**A). We confirmed the RNF126 and RAD50 protein–protein interaction by co‐IP experiments (Figure S3A,B, Supporting Information). Subsequently, we speculated that RNF126 may also interact with other components of the MRN, namely, MRE11 and NBS1. Indeed, we showed that GST‐RNF126 was coimmunoprecipitated by each FLAG‐tagged MRN protein (Figure 3B). We further verified that endogenous RNF126 and MRN proteins interacted (Figure 3C). We also found that RNF126 interacts with ATM and ATR. Interestingly, the interaction between RNF126 and ATR, but not the ATM and MRN complex, was induced by irradiation (Figure 3C). Furthermore, we constructed three truncates of RNF126 according to its domains (Figure 3D) and found that only the N‐terminus of RNF126 interacted with MRE11, NBS1, and ATR (Figure 3E,G and Figure S3E, Supporting Information), whereas both the N‐terminus and the C‐terminus of RNF126 interacted with RAD50 (Figure 3F). In addition, both the N‐ and C‐terminus of RAD50 interacted with RNF126 (Figure S3C,D, Supporting Information). To further validate the dynamic interaction between RNF126 and the MRN complex, we performed protein fractionation experiments by fast protein liquid chromatography (FPLC) in HCC1806 cells treated with IR (10 Gy) and found that native RNF126 co‐eluted with the MRN complex and ATR (Figure 3H). However, the deletion of RNF126 did not affect the assembly of the MRN complex (Figure S3F, Supporting Information). Together, these results demonstrate that RNF126 interacts with the MRN complex and ATR and that the interaction between RNF126 and ATR is significantly enhanced after IR, while RNF126 itself is not involved in the assembly of the MRN complex.

### 2.4 RNF126 Ubiquitinates MRE11 with K27/K29‐Linked Polyubiquitin Chains at K339 and K480

**Figure 4:**
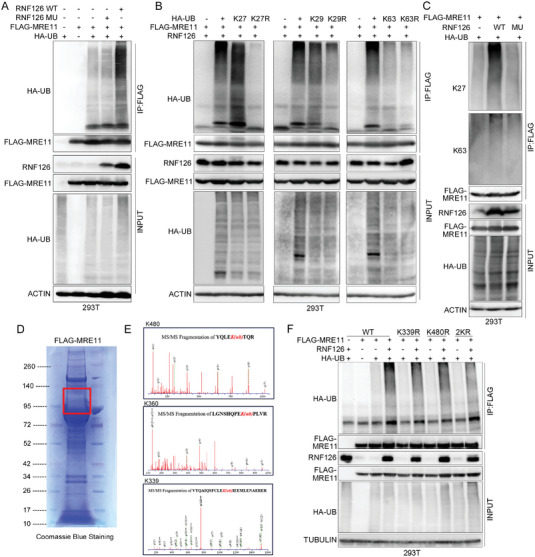
RNF126 ubiquities MRE11 at K339 and K480 and promotes its exonuclease activity. A) RNF126 increased the ubiquitination of MRE11. HEK293T cells expressing FLAG‐MRE11 were co‐transfected with HA‐Ub and RNF126 WT/MU. Cellular extracts were immunoprecipitated with anti‐FLAG followed by in vivo ubiquitination assay analysis of polyubiquitination of MRE11. B) Overexpressing RNF126 did not increase MRE11 ubiquitination in the presence of K27R and K29R Ub. HEK293T cells expressing FLAG‐MRE11 were cotransfected with WT RNF126 and HA‐Ub/K27/K27R, HA‐Ub/K29/K29R, or HA‐Ub/K63/K63R, respectively. Cellular extracts were immunoprecipitated with anti‐FLAG antibody followed by in vivo ubiquitination assay analysis of polyubiquitination of MRE11. C) RNF126 mainly mediates K27‐linked polyubiquitination of MRE11. HEK293T cells expressing FLAG‐MRE11 were co‐transfected with HA‐Ub and RNF126 WT/MU. Cellular extracts were immunoprecipitated with anti‐FLAG antibody followed by in vivo ubiquitination assay analysis of polyubiquitination of MRE11 with antibodies that specifically recognizes K27‐ or K63‐linked polyubiquitin chains. D) Purified FLAG‐MRE11 by immunopurification. Cellular extracts from HEK293T cells expressing FLAG‐MRE11 were immunopurified with anti‐FLAG affinity beads and eluted with FLAG peptide. The eluates were resolved on SDS/PAGE and Coomassie blue staining of purified FLAG‐MRE11. Bands indicated in the red boxes were cut and subjected to mass spectrometry analysis of the ubiquitin site(s). E) K480/K360/K339 were identified as the ubiquitinated residues of MRE11 via mass spectrometric analysis. F) RNF126 promotes polyubiquitination of MRE11 at K339 and K480. HEK293T cells expressing FLAG‐MRE11 or FLAG‐MRE11 K339R/K480R/2KR (containing both K339R and K480R mutation) mutants were co‐transfected with HA‐Ub and RNF126 WT. Cellular extracts were immunoprecipitated with anti‐FLAG affinity beads followed by in vivo ubiquitination assay analysis of polyubiquitination of MRE11.

We then explored the consequence of the physical interaction between RNF126 and the MRN complex. We found that overexpression of RNF126 efficiently increased the ubiquitination of MRE11 but slightly influenced the ubiquitination of NBS1, RAD50, or ATR in an E3 ligase activity‐dependent manner (**Figure** **4**A and Figure S4A,B, Supporting Information).

Although ubiquitination usually targets substrates for degradation, RNF126 did not promote MRE11 degradation (Figure 4A), indicating that RNF126 ubiquitinated MRE11 with a non‐degradative polyubiquitin chain form. Indeed, RNF126 promoted the ubiquitination of MRE11 in the presence of HA‐tagged WT ubiquitin and K27‐only ubiquitin and slightly promoted its ubiquitination in the presence of K29‐only and K0 ubiquitin (Figure S4C, Supporting Information). Furthermore, RNF126 did not increase MRE11 ubiquitination in the presence of K27R and K29R ubiquitin (Figure 4B). In addition, we validated this result by using an antibody that specifically recognizes K27‐ and K63‐linked polyubiquitin chains (Figure 4C, the anti‐K29‐linked polyubiquitin chain Ab is not available). These results indicated that RNF126 mainly mediates K27/K29‐linked polyubiquitination of MRE11.

Then, we used mass spectrometry to identify the ubiquitin site(s) of purified FLAG‐MRE11 (Figure 4D) and found that K480, K360, and K339 might be the ubiquitin site(s) of RNF126 (Figure 4E). The RNF126‐mediated ubiquitination of MRE11 decreased in MRE11 K339R and K480R but not the K360R mutant and more significantly decreased in the K339R/K480R (2KR) mutant (Figure 4F and Figure S4D, Supporting Information). Altogether, our results indicate that RNF126 mainly promotes K27/K29‐linked polyubiquitination of MRE11 at K339 and K480.

### 2.5 RNF126‐Mediated MRE11 Ubiquitination is Required for IR‐Induced ATR‐CHK1 Activation and HR Repair

**Figure 5:**
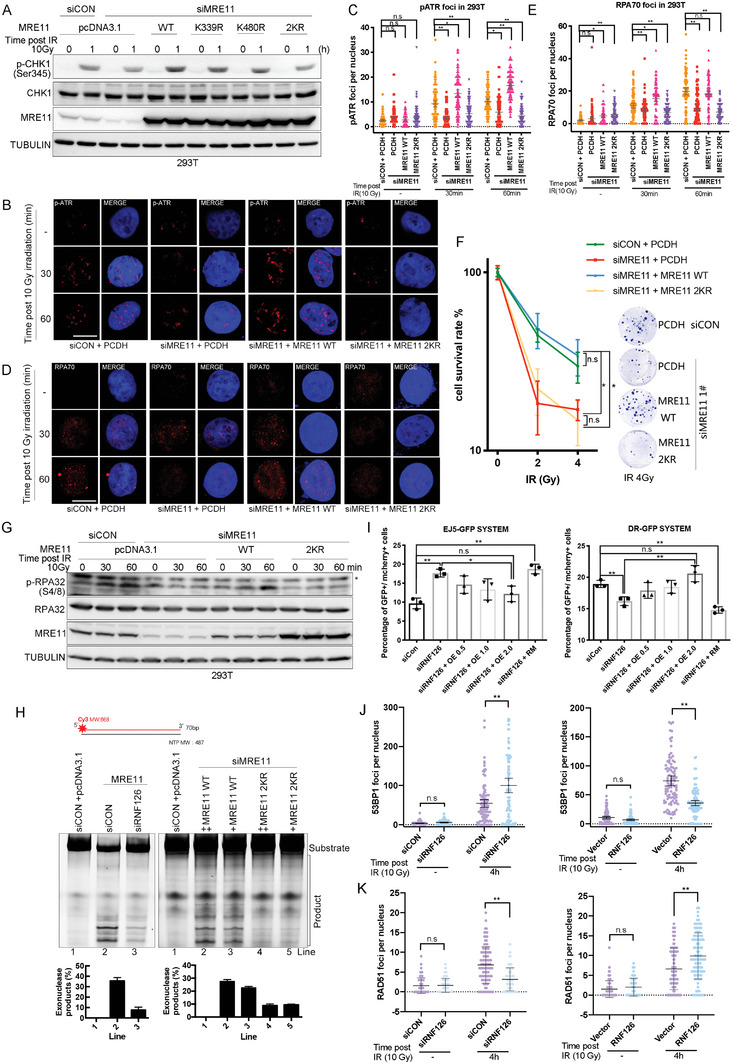
RNF126‐depended MRE11 ubiquitination is required for IR‐induced ATR‐CHK1 pathway activation and HR repair. A) MRE11 knockdown‐impaired CHK1 (p‐Ser345) signals reversed by re‐expressing WT MRE11 but not MRE11 K339/K480/2KR mutants in HEK293T cells. Endogenous MRE11 knockdown HEK293T cells were further overexpressed MRE11 WT or MRE11 K339R/K480R/2KR mutants following treatment with IR (10 Gy). Cell lysates were harvested for Western blotting analysis at 0, 30, and 60 min points post‐IR. B–E) MRE11 knockdown‐impaired IR‐induced ATR (p‐Ser428) and RPA70 foci formation reversed by re‐expressing WT MRE11 but not MRE11 2KR mutants in HEK293T cells. Endogenous MRE11 knockdown HEK293T cells were further overexpressed MRE11 WT or MRE11 K339R/K480R/2KR mutants following treatment with IR (10 Gy). Immunostaining analysis of B,C) p‐ATR and D,E) RPA70 foci formation after irradiation (10 Gy) at 0, 30, and 60 min points. Data are mean ± 95% confidence interval (CI); Statistical analysis was performed using two‐tailed unpaired *t*‐tests. Each point represents a cell. 100 cells quantified in each group were obtained from one experiment. Data are representative of three independent experiments. * *p* < 0.05 and ** *p* < 0.01; n.s, not significant. Scale bars, 10 µm. F) WT MRE11 over‐expression but not MRE11 2KR mutants restored IR‐resistance in MRE11 knockdown HEK293T cells. Clonogenic survival in response to IR of the indicated MDA‐MB‐231 cell lines. Data are mean ± SD. Statistical analysis was performed using two‐tailed unpaired *t*‐tests. Data are representative of three independent experiments. * *p* < 0.05 and ** *p* < 0.01; n.s, not significant. G) Indicated HEK293T cell lines were treated with IR (10 Gy) and harvested for Western blotting analysis at 30 and 60 min points. MRE11 knockdown‐impaired RPA (p‐Ser345) signals were reversed by re‐expressing WT MRE11 but not MRE11 2KR mutants in HEK293T cells. Endogenous MRE11 knockdown HEK293T cells were further overexpressed MRE11 WT or MRE11 2KR mutants following treatment with IR (10 Gy). Cell lysates were harvested for Western blotting analysis at 0, 30, and 60 min points post‐IR. H) RNF126‐mediated ubiquitination of the MRE11 at K339 and K480 sites promotes the exonuclease activity of MRE11. The 5' Cy3‐labeled substrates were incubated with purified MRE11 from indicated HEK293T cells at 37 °C for 1 h. Reactions were stopped by SDS and proteinase K and resolved on 27% denatured polyacrylamide gel. The data are presented as means ± SD. I) RNF126 promotes HR but inhibits NHEJ. RNF126 knockdown EJ5‐GFP‐U2OS or DR‐GFP U2OS cells cotransfected different amounts of RNF126 WT/MU with I‐SCEI plasmid for 48 h and the percentage of GFP+ cells was analyzed by fluorescent activated cell sorting (FACS). Data are mean ± SD. Statistical analysis was performed using two‐tailed unpaired *t*‐tests. Data are representative of three independent experiments. * *p* < 0.05 and ** *p* < 0.01; n.s, not significant. J,K) RNF126 promotes HR but inhibits NHEJ. The quantification of J) 53BP1 or K) RAD51 foci formation after irradiation (10 Gy) at 0 and 4 h in the indicated U2OS cell lines. Data are mean ± 95% confidence interval (CI) Statistical analysis was performed using two‐tailed unpaired *t*‐tests. Each point represents a cell. 100 cells quantified in each group were obtained from one experiment. Data are representative of three independent experiments. * *p* < 0.05 and ** *p* < 0.01; n.s, not significant.

We subsequently sought to determine the role of RNF126‐mediated ubiquitination of MRE11 in the DDR. We first reconfirmed that knockdown (KD) of MRE11 significantly inhibited IR‐induced ATR‐CHK1 pathway activation in MDA‐MB‐231 and HCC1806 cells (Figure S5A, Supporting Information). We found that re‐expression of MRE11 WT effectively rescued IR‐induced activation of CHK1 in MRE11 KD HEK293T cells, whereas re‐expression of MRE11 2KR mutants could not (**Figure** **5**A). Notably, MRE11 K339R or K480R still had a moderate rescue effect on CHK1 activation, suggesting that both K339 and K480 sites play a role in IR‐induced CHK1 activation. Similarly, MRE11 WT, but not MRE11 2KR, efficiently rescued IR‐induced ATR (p‐Ser428) and RPA70 foci formation in MRE11 KD HEK293T cells (Figure 5B–E). Furthermore, we tested the sensitivity of these cells to IR using a colony formation assay. As expected, MRE11 KD increased the sensitivity of HEK293T cells to IR, whereas re‐expressing WT MRE11, but not the 2KR mutant, rescued the phenotype (Figure 5F).

It is well known that MRN complex activation of ATR mainly depends on MRE11 nuclease activity.^[^ ^26^, ^27^ ^]^ In the S/G2 phase, MRE11 cleaves the 5' strand of DNA DSB ends through its endonuclease activity and subsequently generates a single‐stranded DNA gap through its 3' to 5' exonuclease activity, thus initiating ssDNA production and recruiting downstream RPA to ssDNA, activating ATR‐CHK1 signaling.^[^ ^26^, ^27^ ^]^ Therefore, we speculated that RNF126‐dependent ubiquitination at MRE11 K339 and K480 might affect MRE11 3''‐5'' exonuclease activity and subsequent downstream signal cascades. To test this hypothesis, phosphorylated RPA32 (pS4/8), a classical indicator of MRE11 exonuclease activity, was detected by western blot in HEK293T cells. We found that MRE11 KD significantly decreased IR‐induced RPA32 (pS4/8) signaling, which could be mainly recovered by re‐expressing MRE11 WT but not the MRE11 2KR mutant (Figure 5G). We further purified the FLAG‐MRE11 WT or 2KR complex in MRE11 KD HEK293T cells and then incubated them with 5'‐Cy3‐labeled dsDNA substrate. We found that MRE11 WT exhibited stronger nuclease activity than the 2KR mutant (Figure 5H, right and Figure S5B, Supporting Information, right). We also found that the nuclease activity of FLAG‐MRE11 was significantly decreased in RNF126 KD HEK293T cells compared with that in the control group (Figure 5H, left and Figure S5B, Supporting Information, left). These results suggested that RNF126‐mediated ubiquitination of MRE11 at the K339 and K480 sites promotes the exonuclease activity of MRE11.

It is well known that HR‐ and ATR‐mediated checkpoint responses for DSB repair are both initiated by MRE11 nuclease,^[^ ^26^, ^27^, ^28^ ^]^ and the level of activated MRE11 determines the preference of DSB repair by error‐free HR or error‐prone NHEJ.^[^ ^29^ ^]^ Therefore, we tested whether the RNF126‐MER11 axis is involved in directing DSB repair. We employed EJ5‐GFP and DR‐GFP,^[^ ^30^ ^]^ two I‐SceI‐based DSB repair reporter systems that allow quantification of the effect of NHEJ and HR, respectively, in U2OS cells (Figure S5C, Supporting Information). We found that RNF126 KD significantly increased the proportion of NHEJ but decreased the proportion of HR. With the gradual re‐expression of RNF126, the proportion of NHEJ decreased gradually, while the proportion of HR increased gradually. However, re‐expressing the catalytically inactive RNF126 mutant (MU) had no apparent effect on the choice of NHEJ or HR (Figure 5I and Figure S5D, Supporting Information). Furthermore, we detected the foci formation of 53BP1 and RAD51, the key regulators of NHEJ and HR, respectively. Consistently, 53BP1 foci were increased in RNF126 KD cells and decreased in RNF126‐overexpressing cells after IR treatment (Figure 5J and Figure S5E,F, Supporting Information). In contrast, RAD51 foci were decreased in RNF126 KD cells and increased in RNF126‐overexpressing cells after IR treatment (Figure 5K and Figure S5E,G, Supporting Information). Taken together, these data show that RNF126‐dependent MRE11 ubiquitination promotes the activation of ATR‐CHK1 signaling and facilitates HR by promoting the exonuclease activity of MRE11.

### 2.6 RNF126 is Induced by the HER2‐AKT‐NF‐*κ*B Pathway Upon IR

**Figure 6:**
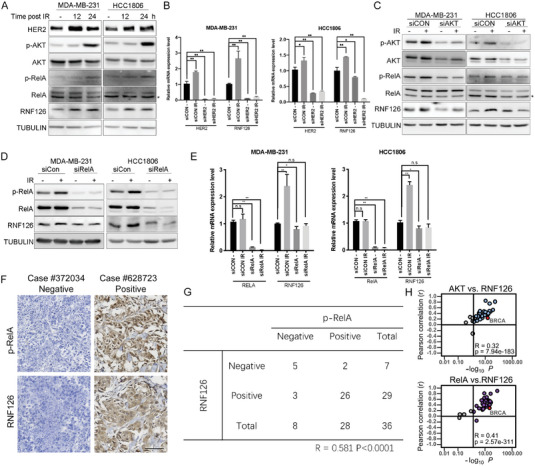
RNF126 expression is induced by IR activating the HER2‐AKT‐NF‐*κ*B pathway. A) IR‐induced RNF126 expression. MDA‐MB‐231 and HCC1806 cell lines were treated with IR (10 Gy) and harvested for Western blotting analysis at 0, 12, and 24 h points post‐IR. B) HER2 knockdown inhibited IR‐induced *RNF126* mRNA expression. MDA‐MB‐231 and HCC1806 transfected with siCON or siHER2 following treatment with IR (10 Gy) and harvested for qPCR analysis at 24 h point post‐IR. C) AKT knockdown inhibited IR‐induced expression of p‐RelA and RNF126. MDA‐MB‐231 and HCC1806 transfected with siCON or siAKT following treatment with IR (10 Gy) and harvested for Western blotting analysis at 0 and 24 h point post‐IR. D) Knockdown RelA inhibited IR‐induced expression of RNF126. MDA‐MB‐231 and HCC1806 transfected with siCON or siRelA following treatment with IR (10 Gy) and harvested for Western blotting analysis at 0 and 24 h point post‐IR. E) Knockdown RelA inhibited IR‐induced *RNF126* mRNA expression. MDA‐MB‐231 and HCC1806 transfected with siCON or siRelA following treatment with IR (10 Gy) and harvested for qPCR analysis at 24 h point post‐IR. F,G) The expression levels of p‐RelA and RNF126 were positively correlated in breast cancer patients receiving radiotherapy. Representative IHC results for p‐RelA and RNF126 are shown. The negative results are shown in the left panel, and the positive results are shown in the right panel. Scale bar, 50 µm. The Spearman's method was used to test the correlation between RNF126 and p‐RelA. H) RNF126 was positively correlated with RelA and AKT in cancer samples. Correlation of RNF126 with RelA and AKT in expression in cancer samples based on the data from the TCGA database. Note that every dot represents one cancer type.

**Figure 7:**
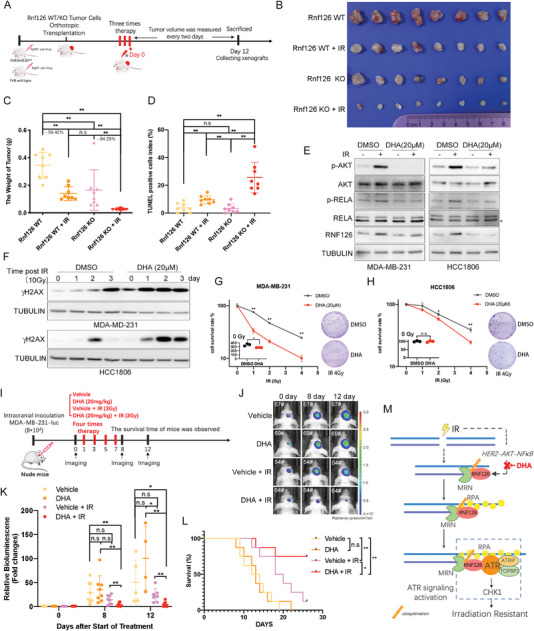
Targeting RNF126 sensitizes TNBC tumors to radiotherapy. A) Schematic diagram of radiotherapy process for PyMT‐induced *Rnf126* WT/KO tumor. B–D) *Rnf126* KO increased the efficacy of radiotherapy. Mice with PyMT‐induced *Rnf126* WT or *Rnf126* KO tumors were divided into two groups, control or radiotherapy (*n* = 4 per group). Twelve days post three times radiotherapy (4 Gy/time), the mice were euthanized; B) The image of tumors was shown, C) the tumors were taken to detect their weight and D) TUNEL positive ratio. Data are mean ± SD. Statistical analysis was performed using two‐tailed unpaired *t*‐tests. * *p* < 0.05 and ** *p* < 0.01; n.s, not significant. E) DHA inhibits RNF126 expression in both MDA‐MB‐231 and HCC1806 cells. MDA‐MB‐231 and HCC1806 cell lines were pretreated with DHA (20 µm) for 2 h before IR (10 Gy) and harvested for Western blotting analysis at 24 h point post‐IR. F) DHA pretreatment increased the accumulation of *γ*H2AX after IR treatment in both MDA‐MB‐231 and HCC1806 cells. MDA‐MB‐231 and HCC1806 cell lines were pretreated with DHA (20 µm) for 2 h before IR (10 Gy) and harvested for Western blotting analysis at 1‐, 2‐, and 3‐day points. G,H) DHA pretreatment decreased survived clones with the increase of IR. Clonogenic survival in response to IR of MDA‐MB‐231 and HCC1806 cell lines pretreated with DHA (20 µm) for 2 h. The column represents the number of clones when only DHA (20 µm) is pretreated. Data are mean ± SD. Statistical analysis was performed using two‐tailed unpaired *t*‐tests. Data are representative of three independent experiments. * *p* < 0.05 and ** *p* < 0.01; n.s, not significant. I) Schematic diagram of characterization of DHA combined with radiotherapy in mice bearing intracranial MDA‐MB‐231 tumors. Cells were engineered to express luciferase. J,K) Combination of DHA and IR treatment inhibited TNBC‐derived metastatic brain tumors with the highest efficiency. Representative images of tumors in the brain imaged by J) IVIS at 0‐, 8‐, and 12‐ day points and the K) fold change of the bioluminescence signal in tumor‐bearing mice received intraperitoneal injection of the vehicle or DHA (20 mg kg^−1^) following with or without irradiation treatment (3 Gy, 4 times) at 0‐, 8‐, and 12‐ day points. Data in J show the mean ± SD. Statistical analysis was performed using the two‐tailed, unpaired Student's *t*‐test. * *p* < 0.05 and ** *p* < 0.01; n.s, not significant. L) Combination of DHA and IR treatment prolonged survival time and survival rate of the mice. Kaplan–Meier survival curves of tumor‐bearing mice receiving the indicated treatments (*n* = 8 per group). Statistical analysis was performed using the log‐rank test. * p < 0.05 and ** p < 0.01; n.s, not significant. M) Model of RNF126 function. We purpose IR‐induced RNF126 expression through HER2‐AKT‐NF‐*κ*B signaling. The increasement of RNF126 induced by IR promotes the exonuclease activity of MRE11 in the case of DSBs, thus promoting ssDNA production and initiating downstream ATR‐CHK1 pathway activation, and ultimately promoting radiotherapy resistance of TNBC cells. Thus, the use of DHA to inhibit IR‐induced RNF126 expression improves the radiotherapy sensitivity of TNBC cells in the brain.

Since RNF126 is involved in the DDR, we wondered whether RNF126 is regulated by IR. We found that RNF126 protein levels were elevated by IR in MDA‐MB‐231 and HCC1806 cells (**Figure** **6**A). We speculated that RNF126 expression was induced by the DDR signaling pathway. In TNBC, IR is known to induce HER2 expression and activate the PI3K‐AKT pathway, which in turn activates the NF‐*κ*B pathway.^[^ ^31^, ^32^, ^33^ ^]^ Indeed, IR induced the expression of HER2, p‐AKT, p‐RelA, and RNF126 (Figure 6A). Therefore, we first tested whether HER2 positively regulates RNF126 expression. As expected, HER2 KD or lapatinib, a HER2 inhibitor, decreased the RNF126 mRNA and protein levels in the HER2‐positive breast cancer cell line BT474 (Figure S6A–C, Supporting Information). Consistently, HER2 KD inhibited IR‐induced *RNF126* mRNA expression in both MDA‐MB‐231 and HCC1806 cells (Figure 6B). In agreement with this, the knockdown of AKT or inhibition of AKT signaling by LY294002 or wortmannin decreased RNF126 mRNA and protein levels in these cell lines (Figure S6D–G, Supporting Information). Importantly, the knockdown of AKT effectively inhibited IR‐induced expression of p‐RelA and RNF126 (Figure 6C). Similarly, the knockdown of RelA effectively inhibited IR‐induced RNF126 mRNA and protein levels (Figure 6D,E). ChIP‐PCR and dual luciferase reporter assay were performed to further demonstrate that IR induced RNF126 transcription through increasing RelA binding to its promoter (Figure S6H–K, Supporting Information). Then, we detected the protein levels of p‐RelA and RNF126 in clinical samples of patients receiving radiotherapy by immunohistochemical staining. Remarkably, a positive correlation (*r* = 0.581, *p* < 0.0001) between p‐RelA and RNF126 was observed in these samples (Figure 6F,G). Moreover, *RNF126* showed a positive correlation with both *RelA* and *AKT* at the mRNA level in most cancer and normal tissues through correlation analysis of The Cancer Genome Atlas (TCGA) database and The Genotype‐Tissue Expression (GTEx) database, respectively (Figure 6H and Figure S6L, Supporting Information). Taken together, these data indicate that IR induces RNF126 expression through HER2‐AKT‐NF‐*κ*B signaling pathways in TNBC cells (**Figure** **7**M).

### 2.7 Inhibition of RNF126 Expression Genetically or Pharmacologically by Dihydroartemisinin (DHA) Sensitizes TNBC to Radiotherapy

Based on the above study, we wondered whether RNF126‐depleted tumors exhibit elevated radiotherapy sensitivity. First, we used the Cre‐PyMT virus to induce breast tumors in *Rnf126* ^fl/fl^ or *Rnf126* wild‐type FVB mice. Then, tumors were digested and transplanted in situ into the breasts of FVB background mice. After low‐dose radiotherapy or mock therapy, the mice were euthanized and the tumors were taken to detect their weight and TUNEL‐positive ratio (Figure 7A). We observed significant growth inhibition of the *Rnf126* KO tumors compared with *Rnf126* WT tumors (Figure 7B and Figure S7A,B, Supporting Information). IR treatment resulted in the weight of tumor reduction rates of 59.42% and 84.29% in the *Rnf126* WT group and *Rnf126* KO group, respectively (Figure 7C). As expected, compared with other groups, *Rnf126* KO tumors in the radiotherapy group had the smallest weight (Figure 7B,C) and the highest proportion of TUNEL‐positive apoptotic cells and signal of *γ*H2AX (Figure 7D and Figure S7C,D, Supporting Information). These results suggested that RNF126 KO increased IR‐induced breast cancer cell apoptosis and the efficacy of radiotherapy.

As RNF126 can be induced by IR through HER2‐AKT‐NF‐*κ*B signaling, we tested whether DHA, a drug that inhibits both the AKT and NF‐*κ*B pathways,^[^ ^34^, ^35^ ^]^ could enhance IR therapy sensitivity of TNBC by inhibiting IR‐induced RNF126 expression. First, we confirmed that DHA efficiently inhibited IR‐induced activation of the AKT‐NF‐*κ*B pathway and RNF126 expression in both MDA‐MB‐231 and HCC1806 cells (Figure 7E). Furthermore, DHA pretreatment sensitized MDA‐MB‐231 and HCC1806 cells to IR treatment, as evidenced by the increased accumulation of *γ*H2AX after IR and fewer surviving clones along with the increased irradiation in vitro (Figure 7F–H). Brain metastases are an important cause of mortality in patients with metastatic breast cancer.^[^ ^36^ ^]^ Since radiotherapy remains the major treatment for brain metastasis of breast cancer, we tested the effect of DHA on IR sensitivity in TNBC tumors in the brain established using MDA‐MB‐231‐luciferase cells (Figure 7I). In order to reduce the damage of IR therapy to normal brain tissue, we chose a lower dose of each treatment (3 Gy/time), while the therapeutic dose remained at 12 Gy in total. Remarkably, the combination of DHA and IR treatment inhibited brain tumor progression with the highest efficiency (Figure 7J,K and Figure S8A,B, Supporting Information) and significantly prolonged the survival time and survival rate of the mice (Figure 7L). Consistently, expression inhibition of RNF126 and severe *γ*H2AX accumulation were detected in the DHA‐IR joint treatment tumors (Figure S8C, Supporting Information). The results proved that pre‐administration of DHA highly improved the therapeutic benefit of IR therapy against TNBC tumors in the brain.

## 3 Discussion

Radiotherapy is one primary therapeutic strategy for TNBC. However, the inevitable normal tissue damage caused by high‐dose irradiation and primary or secondary irradiation resistance often occurs, which is the principal cause of cancer recurrence and poor prognosis.^[^ ^37^ ^]^ It is valuable to develop approaches to sensitize TNBC to radiotherapy. In this study, we identified RNF126 as a prognostic marker and regulator for radioresistance, especially in TNBC. We found that *Rnf126* whole‐body KO mice exhibited significantly decreased radiation tolerance and that high expression of RNF126 in TNBC patients was associated with poor survival. KO of RNF126 significantly impaired IR‐induced ATR‐CHK1 activation, increased *γ*H2AX accumulation, and enhanced the radiosensitivity of TNBC cells. Mechanistically, we found that RNF126 interacts with the MRN complex and ubiquitinates MRE11 at the K339 and K480 sites with K27/K29 polyubiquitin chains. The RNF126‐mediated ubiquitination of MRE11 promotes its exonuclease activity and is required for ATR‐CHK1 signaling activation and HR repair in DDR. In addition, RNF126 expression can be induced by IR through HER2‐AKT‐NF‐*κ*B signaling. More importantly, inhibition of IR‐induced expression of RNF126 by DHA significantly promoted the vulnerability of TNBC cells to IR in vitro and in vivo (Figure 7M). Hence, the IR‐induced RNF126‐MRE11‐ATR‐CHK1 signaling axis may serve as a promising target for improving the efficacy of radiotherapy in TNBC.

Accumulated evidence suggests that RNF126 participates in the DDR in cultured cells. However, the roles of RNF126 in promoting or inhibiting the NHEJ or HR repair pathway remain controversial.^[^ ^18^, ^19^, ^20^, ^21^ ^]^ In this study, we evaluated the role of RNF126 in the DDR regulation in vivo by generating a *Rnf126* whole‐body KO mouse model, a PyMT‐induced primary breast cancer model, and a mouse model with TNBC in the brain. Our results indicate that RNF126 is a possible upstream regulator of HR repair choice (Figure 5I–K). Due to the limitations of the experimental conditions, we did not detect either HR or NHEJ efficiency in one system at the same time. However, several studies have shown that the 3′ to 5′ exonuclease activity of MRE11 determines how cells repair DNA DSBs. Therefore, it is reasonable to conclude that RNF126, as a regulator of MRE11 exonuclease activity, determines the direction of DNA damage repair during DSBs and thus determines the fate of cells. This is consistent with the results obtained in animal experiments in which *Rnf126*‐deficient mice showed worse radiation tolerance during whole‐body irradiation. When DSBs occurred after RNF126 deficiency, the cells were more inclined to be error‐prone NHEJs for DDR. Thus, we suspect that *Rnf126* KO mice harbor more genome instability and increased cancer susceptibility. These speculations need further investigation in the future.

MRE11 binds damaged DNA and nucleolytically processes the resection of DNA ends, a function that is key to ATR‐CHK1 activation and initiates HR but inhibits the NHEJ repair pathway.^[^ ^27^, ^38^ ^]^ Deficiency of the *MRE11* gene causes diseases related to genomic instability, such as ataxia‐telangiectasia‐like disorder (ATLD).^[^ ^39^ ^]^ Several rare MRE11 mutations in patients were identified to be related to higher breast cancer susceptibility.^[^ ^40^ ^]^ Although MRE11 has previously been described as an important factor for DNA repair, its ubiquitination is largely unknown.^[^ ^29^, ^41^ ^]^ In this study, we showed that MRE11 undergoes RNF126‐dependent K27/K29‐linked polyubiquitination at the K339 and K480 sites, which does not trigger MRE11 degradation but promotes its exonuclease activity with no effect on MRN complex assembly. Previous studies have found that the PRMT1‐mediated arginine methylation of MRE11 regulates its 3′ to 5′ exonuclease activity.^[^ ^42^ ^]^ How ubiquitination increases MRE11 exonuclease activity also needs further investigation.

Our results indicate that the RNF126‐MRE11 axis contributes to ATR activation. Compared to ATM, ATR usually copes with wider DNA damage problems and replication stress to maintain genomic integrity and cell survival.^[^ ^17^, ^18^ ^]^ As anti‐tumor factors, genes in the ATM pathway frequently lose copies or are mutated in cancer cells, whereas genes in the ATR pathway are rarely mutated.^[^ ^19^, ^20^, ^21^, ^22^, ^23^, ^24^ ^]^ Consequently, the ATR pathway may be particularly important for tumor cell survival, such as TNBC, which is characterized by the deficiency of the DDR pathway because of mutations of DDR genes, including *BRAC1/2*, *p53*, and *ATM*.^[^ ^22^ ^]^ Inhibition of ATR can be considered a potential therapy.^[^ ^33^, ^34^, ^35^ ^]^ In this study, we found that RNF126 promotes IR‐induced specific activation of ATR‐CHK1, but not ATM‐CHK2. Two previous studies reported on RNF126 and ATR‐CHK1 signaling activation,^[^ ^18^, ^43^ ^]^ however, the in‐depth mechanism has not been investigated. In this study, we elaborated on the mechanism by which RNF126 acts on the activation of ATR‐CHK1 through the ubiquitination of MRE11 to promote its exonuclease activity.

To date, only the ERK pathway has been reported as the upstream regulator of RNF126.^[^ ^44^ ^]^ Here, for the first time, we found that IR promoted the transcription of *RNF126* by activating the HER2‐AKT‐NF‐*κ*B pathway. Previous studies have shown that in breast cancer cells, IR‐stimulated HER2‐mediated PI3K‐AKT signaling activates NF‐*κ*B to induce RelA‐mediated HER2 transcription, which further boosts AKT‐NF‐*κ*B signaling.^[^ ^31^, ^45^ ^]^ Moreover, IR also activated HER2‐ERK signaling. However, we found that AKT pathway inhibitors exhibited dominant inhibition of RNF126 expression in several breast cancer cell lines (data not shown). Here, we found that RelA binding to the *RNF126* promoter responds to IR‐induced NF‐*κ*B pathway activation. It is worth noting that, HER2 is possibly necessary but not sufficient to induce RNF126 expression. RNF126 expression was not higher in HER2‐positive breast cancers than in other types of breast cancer and their expressions were not correlated in breast cancer (Figures S1E and S6M, Supporting Information). We suspect that the expression of RNF126 mainly depends on the activation of both AKT and NF‐*κ*B signaling pathways (Figure 6 and Figure S6, Supporting Information). In TNBC, IR stimulates HER2 expression to further activate AKT and NF‐*κ*B pathways, thereby boosting the expression of RNF126. However, in HER2‐positive breast cancer cells, the AKT and NF‐*κ*B pathways may not be activated simultaneously because of other regulations. In addition, it cannot be ruled out that IR‐induced RNF126 expression may require other components other than the activated HER2/AKT/NF‐*κ*B axis.

Several studies have reported that DHA has a radiosensitizing effect on lung cancer, colorectal cancer, and glioma cells.^[^ ^46^, ^47^ ^]^ Meanwhile, there are several phases I to IV clinical studies on DHA for malaria and lupus erythematosus. Due to its good oral absorption, few side effects, and high efficacy, DHA has great potential for clinical application and may become a valuable choice for first‐line treatment to increase tumor radiotherapy sensitivity. In addition, DHA has been reported a potential role in immunotherapy due to its immune‐modulatory properties.^[^ ^48^ ^]^ And accumulating evidence shows that radiotherapy can reprogram the tumor microenvironment, converting tumors from immunogenic “cold” to “hot”, and vulnerable to the impact of immue checkpoint blockade therapy.^[^ ^49^ ^]^ It is worth further exploring whether DHA can be jointly used to enhance the efficacy of immunotherapies while increasing the sensitivity of radiotherapy. Furthermore, consistent with our study, AKT inhibitors and HER2 inhibitors have also been reported to increase radiotherapy sensitivity in a variety of cancers.^[^ ^33^, ^50^, ^51^ ^]^ However, inhibitors targeting RNF126 have not been developed. We are currently screening RNF126 inhibitors and will test their potential for radiotherapy sensitization.

In summary, our study reveals that RNF126‐mediated MRE11 ubiquitination is a critical regulator of the DDR. Based on our findings, we propose a working model to demonstrate the mechanism by which RNF126 activates ATR through MRE11 during DSBs (Figure 7M). Upon encountering DSBs, RNF126 interacts with MRE11 and induces mainly K27/K29‐linked polyubiquitination of MRE11 at its K339 and K480 sites, thus promoting the 3′ to 5′ exonuclease activity of MRE11 to generate more ssDNA, resulting in the recruitment of RPA and its downstream complex and finally activating ATR‐CHK1 signaling. Our findings reveal molecular insight into how the IR‐HER2/AKT/NF‐*κ*B‐RNF126‐MRE11‐ATR/CHK1 pathway is activated and how DDR choices are determined in cells during DSBs, in turn providing innovative paradigms for the DNA damage response and therapeutic applications for cancer treatment.

## 4 Experimental Section

### Cell Culture and Treatment

HCC1806, MDA‐MB‐231, HEK293T, and U2OS cells were purchased from American Type Culture Collection (Manassas, VA, USA) and validated via short tandem repeat analysis. The DR‐GFP‐U2OS and EJ5‐GFP‐U2OS cell lines were gifts from Prof. Junmin Zhou from the Kunming Institute of Zoology, Chinese Academy of Sciences.^[^ ^52^ ^]^ HCC1806 cells were cultured in Roswell Park Memorial Institute‐1640 medium containing 5% fetal bovine serum. MDA‐MB‐231, U2OS, and HEK293T cells were grown in Dulbecco's modified Eagle's medium supplemented with 10% fetal bovine serum. All cells were maintained at 37 °C in an incubator with 5% CO_2_. Dihydroartemisinin (Topscience T0607, China), HER2 inhibitor lapatinib (HY‐50898, MCE, Shanghai, China), AKT inhibitors LY294002 (HY‐10108, MCE, Shanghai, China), and wortmannin (HY‐10197, MCE, Shanghai, China) were diluted in DMSO prior to addition to the culture medium. Cells were irradiated using an Xstrahl CIX3 irradiator (400 mm focus to skin distance with 0.7‐mm filter, 300 kV, 10 mA, dose rate 2.06 Gy min^−1^).

### Plasmids, siRNAs, and Transfection

All transfections for plasmids and siRNAs were performed using Lipofectamine 2000 (Invitrogen, California, USA) according to the manufacturer's instructions. In brief, cells were grown to 50–60% confluence and transfected with the respective plasmids or siRNA. Plasmids or diluted siRNA and Lipofectamine 2000 were added to separation tubes containing serum‐free medium and incubated at room temperature for 5 min. The contents of the two tubes were then mixed, incubated at room temperature for 20 min, and distributed onto the respective cell culture dishes. The cells were incubated in 5% CO_2_ at 37 °C for 48 h for further experiments. All chemically synthesized siRNAs were purchased from RiboBio (Guangzhou, China) and transfected at a final concentration of 20 nM. The siRNA target sequence for the human RNF126 3′‐UTR was 5′‐GUCUAACCUCACCCUCUAA‐3′. The siRNA target sequences for the human MRE11 gene were 5′‐GAACACTAGTTCTTTAAGA‐3’ (1#) and 5′‐GTACGTCGTTTCAGAGAAA‐3’ (2#). The siRNA target sequence for the human AKT gene was 5′‐GUCUAACCUCACCCUCUAA‐3′. The siRNA target sequence for the human RelA gene was 5′‐GCCCUAUCCCUUUACGUCA‐3′. The siRNA target sequence for the human IKK*α* gene was 5′‐GCAGGCUCUUUCAGGGACA‐3′. The plasmids used in this study were constructed by Mut Express MultiS Fast Mutagenesis Kit (Vazyme C215, China) and Uniclone One Step Seamless Cloning Kit (Genesand SC612, China), and listed in Table S1, Supporting Information.

### Generation of RNF126 KO Cell Lines

Specific CRISPR guides were designed for DNA sequences within the CDS (1#: AGATATAATCCGGCAGGCGC; 3#: AGGCGTCGCCGCATCCCGGA) of the RNF126 gene using the online CRISPR Design Tool (http://tools.genome‐engineering.org). After the target sequence was cloned into lentiCRISPRv2, the packaging plasmids (psPAX2 and pMD2.G) were cotransfected with targeted gene sgRNA‐linked lentiCRISPRv2 into HEK293T cells to prepare lentivirus. Virus‐containing supernatants were harvested 48 and 72 h posttransfection. MDA‐MB‐231 and HCC1806 cells were plated and infected with lentivirus. After 48 h, a fresh medium containing 1 µg mL^−1^ was replaced every 2 days until all control cells died. RNF126 knockout was confirmed by Western blotting.

### Western Blotting and Antibodies

The Western blotting assay was performed according to the protocol described in our previous studies.^[^ ^53^ ^]^ In brief, protein samples were mixed with 1 × SDS buffer (60 mm Tris‐HCl (pH 6.8), 1% (wt/vol) SDS, 5% (vol/vol) glycerol, 0.005% (wt/vol) bromophenol blue, and 1% (vol/vol) 2‐mercaptoethanol) at 98 °C for 10 min, separated by SDS–PAGE, and transferred to PVDF membranes (Millipore, Germany). After blocking with 5% nonfat milk in PBS (LFPBS10L, Lufei, China) with 0.1% Tween 20 (A100777, Sangon Biotech, Shanghai, China), the membranes were incubated with the indicated antibodies overnight at 4 °C, followed by incubation with a horseradish peroxidase‐conjugated secondary antibody for 1 h at room temperature. The protein bands were detected with Super ECL plus (UE, S6009) by ImageQuant LAS4000 (GE, Germany). A list of the antibodies used in our study is provided in Table S2, Supporting Information.

### Ubiquitination Assays

Cells with different treatments were lysed in lysis buffer containing 50 mm Tris‐HCl (pH 7.4), 1 mm EDTA, 1% SDS, 10 mm N‐ethylmaleimide (NEM) and protease inhibitor on ice for 30 min with rotation and centrifuged at 12 000 rpm for 10 min followed by tenfold dilution with BSA buffer (50 mm Tris‐HCl (pH 7.4), 0.5% Nonidet P‐40, 0.5% SDS, 0.5% BSA, 10 mm NEM and protease inhibitor). Approximately 0.5–1.5 mg of cellular extracts were immunoprecipitated with anti‐FLAG agarose affinity gel (A2020, Sigma–Aldrich, St. Louis, MO) for 4 h at 4 °C. The beads were then washed five times with BSA buffer, boiled in SDS loading buffer, and subjected to SDS–PAGE followed by immunoblotting.

### HR and NHEJ Reporter Assays

HR efficiency was examined with DR‐GFP U2OS cells, in which two incomplete copies of GFP genes were integrated into chromosomal DNA and cleavage of the I‐SceI sites led to the restoration of the GFP gene through HR, while NHEJ efficiency was determined with EJ5‐GFP U2OS cells, in which excision of the two I‐SceI sites followed by NHEJ eliminated the translation start codon of the otherwise nonsense transcript and enabled the reading frame shift and subsequent expression of the *GFP* gene. Endogenous RNF126 in DR‐GFP‐/EJ5‐GFP‐ U2OS cells was first knocked down using RNF126 3'UTR siRNA. 24 h after siRNA transfection, the RNF126 WT/MU overexpression plasmid was transfected into cells, and 24 h after plasmid transfection, the I‐SceI plasmid and mCherry plasmid were cotransfected into cells. 48 h after transfection, the percentage of GFP‐positive and mCherry‐positive cells were counted by FACS analysis with Accuri C6 (BD Biosciences). Control group cells were transfected with siCON and the corresponding empty overexpression plasmid. For each treatment, a minimum of 20 000 cells were analyzed by FACS. Data analysis was done using Flowjo software. The effects of knockdown and overexpression were confirmed by Western blotting.

### DNA Substrate

Fluorescence‐labeled 5' overhang DNA substrate, purchased from Sangon Biotech, was prepared by annealing the synthetic oligonucleotides below.

Oligo 1 (70 nt) with modified Cy3 fluorescence dye at the 5' end: 5'‐Cy3‐ GTAAGTGCCGCGGTGCGGGTGCCAGGGCGTGCCCTTGGGCTCCCCG GGCGCGTACTCCACCTCATGCATC ‐3';

Oligo 2 (70 nt) without Cy3 fluorescence dye modified: 5'‐GATGCATGAGGTGGAGTACGCGCCCGGGGAGCCCAAGGGCACGCCC TGGCACCCGCACCGCGGCACTTAC ‐3'

Briefly, equal amounts of oligonucleotides (oligo 1 + 2) were mixed in the annealing buffer (50 mm Tris pH 7.5, 10 mm MgCl_2_, 100 mm NaCl, and 1 mm DTT) and heated at 95 °C for 4 min, followed by removing it to room temperature and allowing the reaction to cool to room temperature for 5–10 min.

### Nuclease Reactions

To evaluate the effect of RNF126 and K339 and K480 sites within MRE11 on MRE11 exonuclease activity, reactions were set up by adding 2 µL FLAG‐MRE11 WT/2KR purified from the indicated HEK293T whole‐cell extracts in 12 µL of reaction buffer (25 mm Tris‐HCl, pH 7.5, 2 mm MnCl_2_, 1 mm DTT, and 100 mg mL^−1^ BSA) containing 100 mm KCl (final concentration), followed by the addition of 500 nM substrate 5’‐end‐Cy3‐labbled dsDNA. After a 90‐min incubation at 37 °C, the reaction was terminated by treatment with 0.5 µL 10% SDS, 0.5 µL 0.5 m EDTA, 0.5 µL proteinase K, and incubation at 50 °C for 30 min. After adding an equal volume of 2 × loading buffer (95% formamide, 20 mm EDTA, 0.01% bromophenol blue), the reaction mixtures were held for 4 min at 95 °C before being analyzed in a denaturing polyacrylamide gel containing 7 m urea in TBE buffer.

### Mice

C57BL/6 *Rnf126* ^fl/fl^ conditional knockout mice were generated by Biocytogen. The 5'‐ and 3'‐end loxP sites were inserted into intron 1 and intron 8 of *Rnf126*, respectively. When Cre was expressed, structural exons 2–8 of the Rnf126 gene could be conditionally knocked out to produce a truncated protein consisting of 74 amino acids (25 aa from the N‐terminus and 49 aa generated by frameshift mutations), triggering nonsense‐mediated mRNA degradation. *Rnf126* ^fl/fl^ mice were crossed with C57BL/6 CMV‐Cre transgenic mice to produce *Rnf126* knockout mice (*Rnf126* KO). Chimeric offspring were backcrossed to FVB mice for 13 generations. All mice were kept in specific pathogen‐free conditions within the Animal Resource Center at the Kunming Institute of Zoology.

### Intracranial Mouse Xenografts

To establish intracranial MDA‐MB‐231‐luc mouse xenografts, 5‐ to 6‐week‐old female nude mice were anesthetized via intraperitoneal injection of xylazine. 80 000 MDA‐MB‐231‐luc cells in 4 µL of PBS were injected into the right striatum 2.5 mm lateral and 0.5 mm posterior to bregma and 3 mm below the dura using a stereotactic apparatus (Rwdls, Shenzhen, China) with an Integrated Stereotaxic Injector (Stoelting, USA). The mice were treated ten days after receiving the injection. After treatment, the mice were imaged by using an IVIS Lumina III imaging system (Xenogen, USA). To alleviate unnecessary pain and distress, the endpoint of the experiment was determined if the body weight of the mice decreased continuously to about 10% of the weight before the experiment or if they were unable to take food or water. Mice were purchased from Charies River (Beijing, China). All animal experiments were performed according to the institutional ethical guidelines of animal care (SMKX‐20160305‐08).

### Statistics and Reproducibility

All statistical analyses were performed using Prism v.9.0 (GraphPad) or Microsoft Excel 2019. Statistical analysis was performed using two‐tailed Student's *t‐*tests unless mentioned otherwise. *p* < 0.05 was considered to be significant. The number of cells, mice or replicates (*n*) for each experiment is indicated in the figures or figure legends. For bar and line graphs, data are presented as the mean ± SD unless specified in the legends. Immunofluorescence micrographs are representative of three independent experiments in the indicated cells of the same treatment. Western blotting data were from the respective experiment, processed in parallel, and are representative of three independent experiments unless specified in the legends.

## Conflict of Interest

The authors declare no conflict of interest.

## Author Contributions

C.C., D.J., and X.Z. supervised the experiments and analyzed data; W.L. designed and performed most experiments; W.L., D.J., and C.C. wrote the draft manuscript. M.Z., R.Z., Q.J., F.L., W.L., L.W., J.W., R.L., Y.G., and X.H. helped with the animal experiments. C.Y. and Y.W. performed the immunohistochemical staining and scoring. W.L. helped with the irradiation assay. R.Z. and M.Z. helped with western blotting. L.S. and K.Z. helped with Immunopurification and LC‐MS/MS. All authors discussed and approved the final manuscript.

## Supporting information

Supporting Information

## Data Availability

This study did not contain high through‐put data. The data resource and materials are available from the corresponding author upon reasonable request.
