# Correction to “RNF126‐Mediated MRE11 Ubiquitination Activates the DNA Damage Response and Confers Resistance of Triple‐Negative Breast Cancer to Radiotherapy”

**DOI:** 10.1002/advs.202417701

**Published:** 2025-01-30

W. Liu, M. Zheng, R. Zhang, Q. Jiang, G. Du, Y. Wu, C. Yang, F. Li, W. Li, L. Wang, J. Wu, L. Shi, W. Li, K. Zhang, Z. Zhou, R. Liu, Y. Gao, X. Huang, S. Fan, X. Zhi, D. Jiang, C. Chen, RNF126‐Mediated MRE11 Ubiquitination Activates the DNA Damage Response and Confers Resistance of Triple‐Negative Breast Cancer to Radiotherapy. *Adv. Sci*. 2023, 10, 2203884. https://doi.org/10.1002/advs.202203884

In the Supplementary Data of the original publication, the image representing the 53BP1 foci in the RNF126 overexpression group was incorrectly presented in Supplementary Figure 5F (right). The correct image should be as follows:


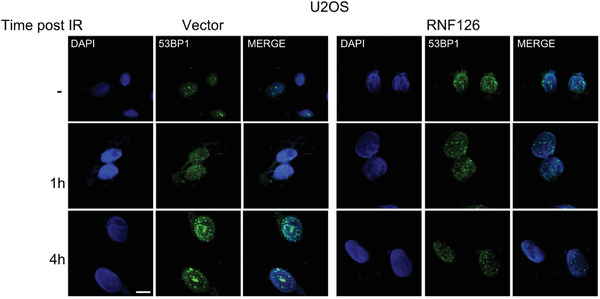


We apologize for this error.

