# Supplementary material for: RNF126‐Mediated MRE11 Ubiquitination Activates the DNA Damage Response and Confers Resistance of Triple‐Negative Breast Cancer to Radiotherapy
Source: Adv Sci (Weinh). 2022 Dec 23;10(5):2203884. doi: 10.1002/advs.202203884 (PMC9929257; doi:10.1002/advs.202203884)
Supplement: Supplementary file 1 — Supporting Information [file ADVS-10-2203884-s001.pdf]

ADVANCED SCIENCE
Open Access

Supporting Information

for *Adv. Sci.*, DOI 10.1002/advs.202203884

RNF126-Mediated MRE11 Ubiquitination Activates the DNA Damage Response and Confers Resistance of Triple-Negative Breast Cancer to Radiotherapy

Wenjing Liu, Min Zheng, Rou Zhang, Qiuyun Jiang, Guangshi Du, Yingying Wu, Chuanyu Yang, Fubing Li, Wei Li, Luzhen Wang, Jiao Wu, Lei Shi, Wenhui Li, Kai Zhang, Zhongmei Zhou, Rong Liu, Yingzheng Gao, Xinwei Huang, Songqing Fan, Xu Zhi, Dewei Jiang* and Ceshi Chen**

Supplementary Materials for:

RNF126-mediated MRE11 ubiquitination activates the DNA damage response and confers resistance of triple-negative breast cancer to radiotherapy

Wenjing Liu[1,2], Min Zheng[1,2], Rou Zhang[1], Qiuyun Jiang[1,2], Guangshi Du[1,2], Yingying Wu[3], Chuanyu Yang[1,2], Fubing Li[4], Wei Li[1,2], Luzhen Wang[1, 5], Jiao Wu[6], Lei Shi[7], Wenhui Li[8], Kai Zhang[7], Zhongmei Zhou[1], Rong Liu[1,9], Yingzheng Gao[10], Xinwei Huang[10], Songqing Fan[11], Xu Zhi[12*], Dewei Jiang[1,2 *], Ceshi Chen[1,13,14 *]

[1] *Key Laboratory of Animal Models and Human Disease Mechanisms of the Chinese Academy of Sciences and Yunnan Province, Kunming Institute of Zoology, Chinese Academy of Sciences, Kunming, China*

[2] *Kunming College of Life Sciences, University of the Chinese Academy of Sciences*

[3] *Department of the Pathology, First Affiliated Hospital of Kunming Medical University, Kunming, Yunnan 650032, China*

[4] *Affiliated Cancer Hospital & Institute of Guangzhou Medical University, Guangzhou, 510095, China*

[5] *School of Life Science, University of Science & Technology of China, Hefei, 230027, Anhui, China*

[6] *Department of the Second Medical Oncology, The Third Affiliated Hospital of Kunming Medical University, Kunming, Yunnan Province, China*

[7] *Tianjin Medical University, Tianjin 300070, China*

[8] *Yunnan Tumor Hospital, The Third Affiliated Hospital of Kunming Medical University, Kunming, Yunnan Province, China*

[9] *Translational Cancer Research Center, Peking University First Hospital, Beijing, 100034, China*

[10] *Department of the Central Laboratory, Second Affiliated Hospital of Kunming Medical University, Kunming, Yunnan 650032, China*

[11] *Department of Pathology, the Second Xiangya Hospital, Central South University, Changsha, Hunan, 410000, China*

[12] *Center for Reproductive Medicine, Department of Obstetrics and Gynecology, Peking University Third Hospital, Beijing, 100191, China*

[13] *Academy of Biomedical Engineering, Kunming Medical University, Kunming 650500, China*

[14] *The Third Affiliated Hospital, Kunming Medical University, Kunming 650118, China*

Correspondence to chenc@mail.kiz.ac.cn, jiangdewei@mail.kiz.ac.cn or zhixujp@163.com

This file includes:

- **Supplementary Figures S1-S8 (with legends)**
- **Supplementary Experimental Section**
- **Supplementary References**
- **Table S1 and S2**

Supplementary Figures

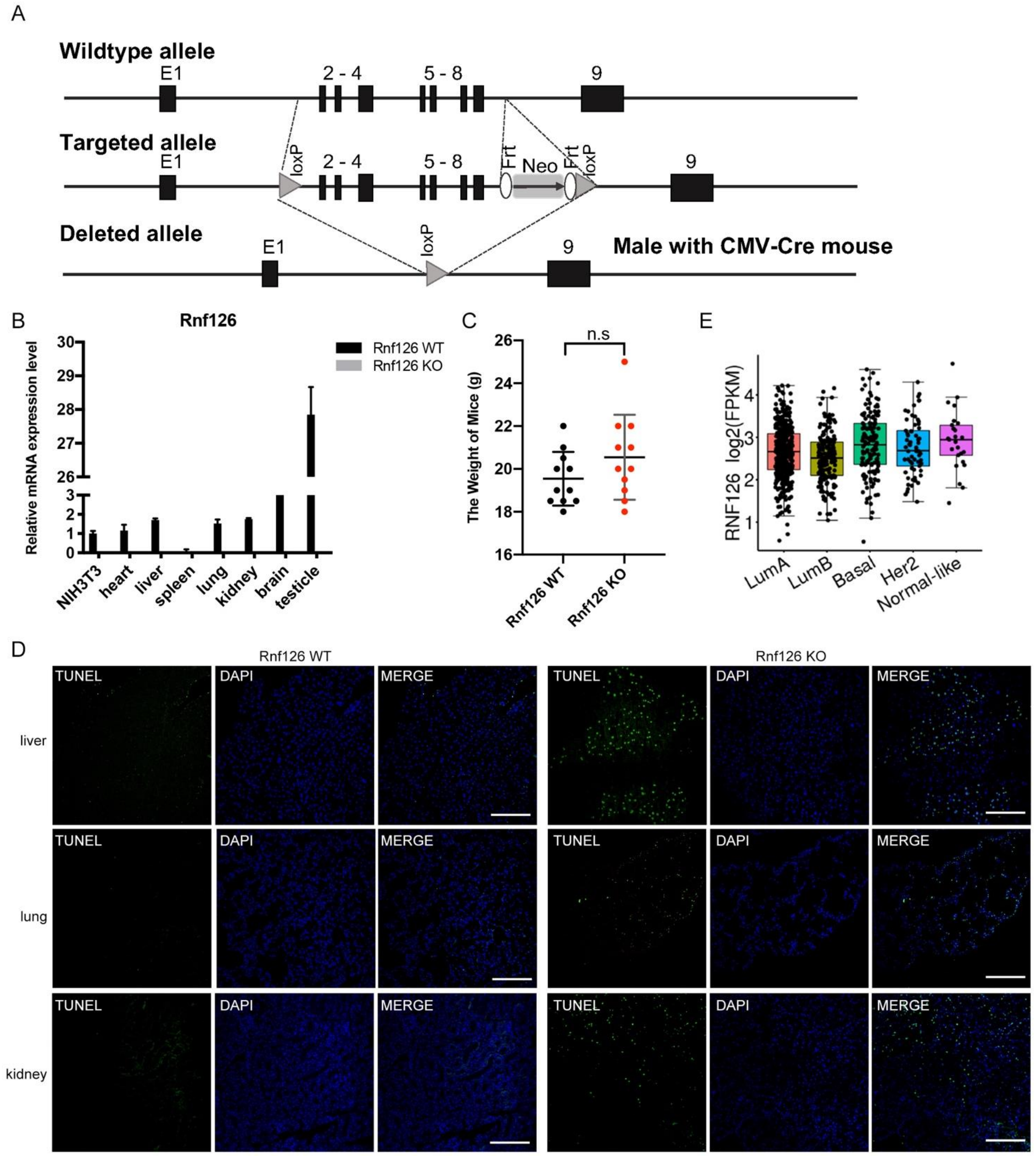

Supplementary Figure 1. Knockout efficiency validation and body weight of RNF126 whole-body knockout mice. A) Targeting strategy used to generate *Rnf126* KO mice (Biocytogen). B) *Rnf126* was not detected at mRNA level in *Rnf126* KO mice tissues. Rnf126 expression level in Rnf126 WT or Rnf126 KO mice measured by RT-qPCR. Data are mean ± s.d. C) *Rnf126* KO had no significant effect on body weight of mice. The weight of Rnf126 WT and Rnf126

KO mice. Data are mean ± s.d. Statistical analysis was performed using two-tailed unpaired *t*-test (*n*=3 mice per genotype). * $p < 0.05$ and ** $p < 0.01$; n.s, not significant. D) The apoptotic cells in the tissues of *Rnf126* whole-body KO mice increased significantly after irradiation. Representative images of TUNEL staining of livers, lungs and kidneys isolated from 3 pair male mice receiving the indicated treatment. Scale bar, 100 μm. E) Analysis of the mRNA expression of *RNF126* in the indicated breast cancer types in the TCGA database.

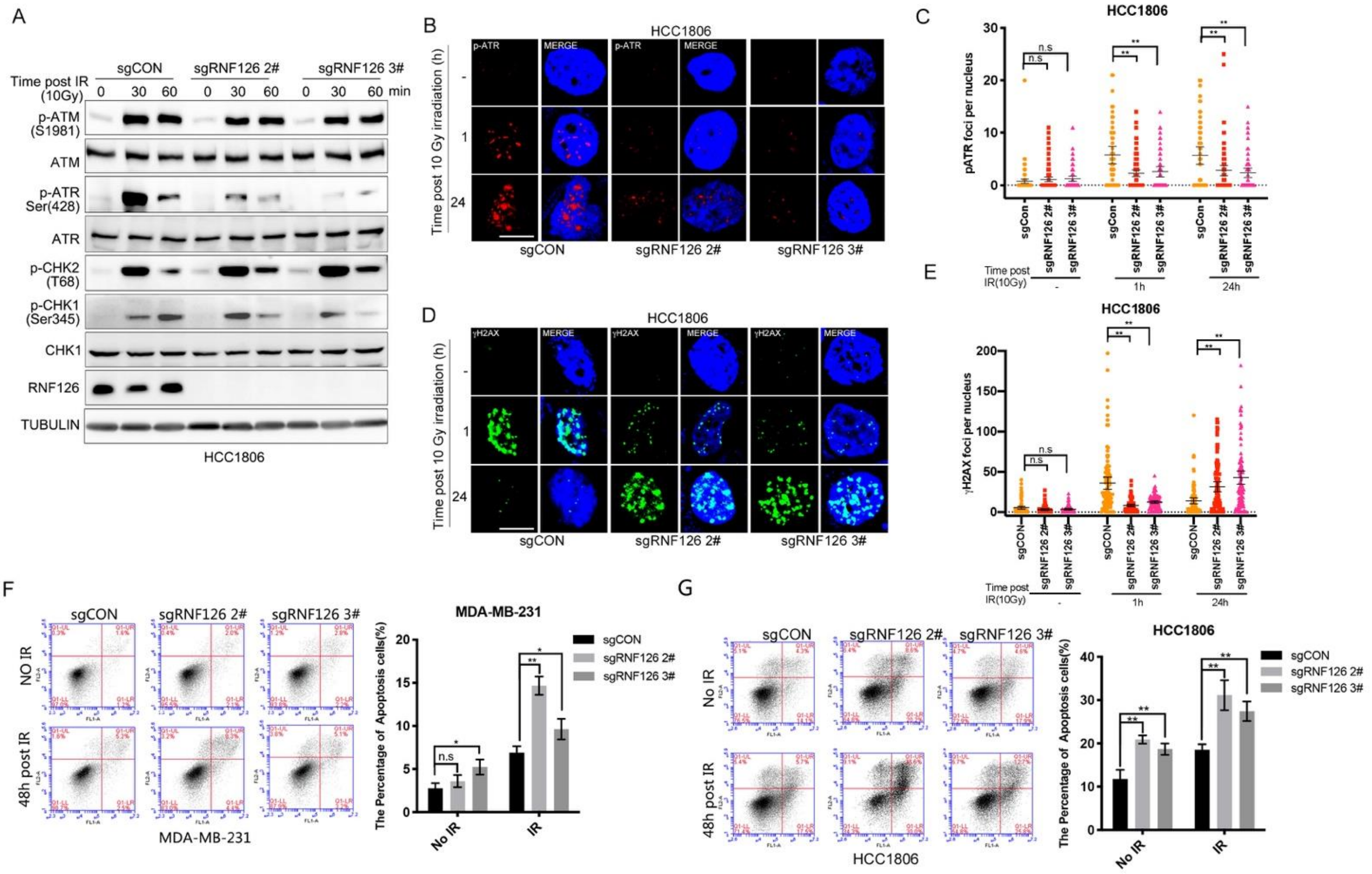

Supplementary Figure 2. RNF126 promotes IR-induced ATR-CHK1 signaling activation depends on its E3 ligase activity. A) RNF126 KO decreased the ATR-CHK1 activation post irradiation in HCC1806 cells. RNF126 stable KO HCC1806 cells treated with IR (10 Gy) and cell lysates were harvested at 0, 30 and 60 min for Western blotting analysis. B,C) *RNF126* KO decreased IR-induced p-ATR foci formation in HCC1806 cells. Immunostaining analysis of p-ATR foci formation after irradiation (10 Gy) at 0, 1 and 24 h in RNF126 stable KO HCC1806 cells followed by quantification. Data are mean ± 95% confidence interval (CI) Statistical analysis was performed using two-tailed unpaired *t*-tests. Each point represents a cell. 100 cells quantified in each group were obtained from one experiment. Data are representative of three independent experiments. * $p < 0.05$ and ** $p < 0.01$; n.s, not significant. Scale bars, 10 μm. D,E) *RNF126* KO increased IR-induced γH2AX accumulation in HCC1806 cells. Immunostaining analysis of γH2AX foci formation after irradiation (10 Gy) at 0, 1 and 24 h in *RNF126* stable KO HCC1806 cells followed by quantification. Data are mean ± 95% confidence interval (CI) Statistical analysis was performed using two-tailed unpaired *t*-tests. Each point represents a cell. 100 cells quantified in each group were obtained from one experiment. Data are representative of three independent experiments. * $p <$ 0.05 and ** $p < 0.01$; n.s, not significant. Scale bars, 10 μm. F,G) The percentage of IR-induced apoptosis in *RNF126* stable KO cells was significantly higher than that in control group. Representative and

quantification graphs of RNF126 stable KO MDA-MB-231 and HCC1806 cell lines treated with or without IR (6 Gy). 48 hours after IR treatment, the cells were harvested for apoptosis assay. Results are presented as mean ± s.d. using two-tailed unpaired *t*-tests, n=3. Data are representative of three independent experiments. * $p < 0.05$ and ** $p < 0.01$; n.s, not significant.

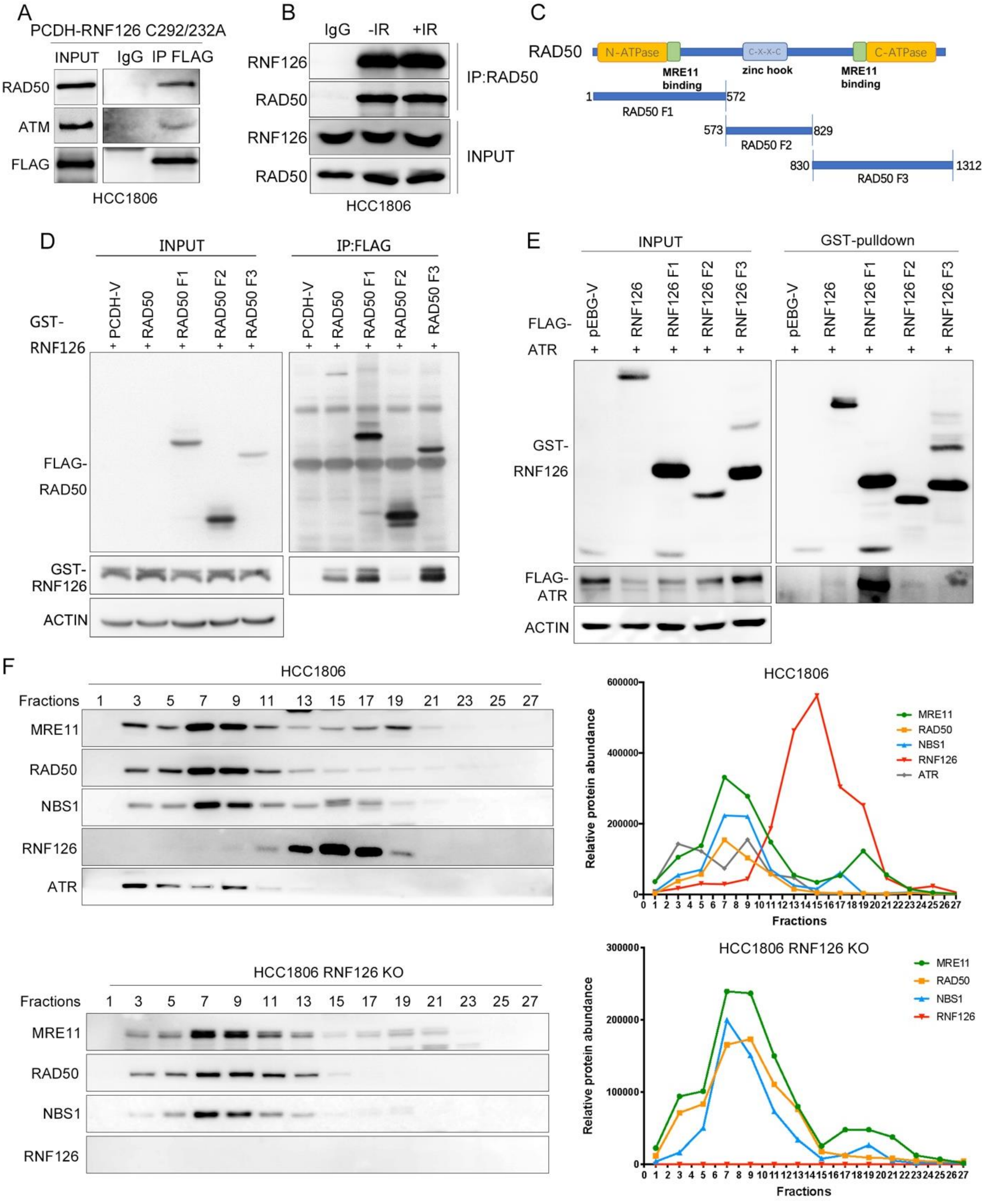

Supplementary Figure 3. RNF126 is physically associated with MRN and ATR. A) Exogenous RNF126 interacted with RAD50. HCC1806 cells stably expressing FLAG-RNF126 C292/232A and whole-cell lysates were collected for co-IP analysis. B) Endogenous RAD50 interacted with RNF126. Co-IP analysis of the association between RNF126 and RAD50. Whole-cell lysates from HCC1806 cells were immunoprecipitated and then immunoblotted with antibodies against the indicated proteins. C) Schematic of RAD50 fragments.

D) Both the N-terminus and C-terminus of RAD50 interacted with RNF126. Map of RNF126 interaction domains of RAD50. GST fused RNF126 and different FLAG-RAD50 fragments were expressed in HEK293T cells, and whole-cell lysates were collected for co-IP analysis. E) N-terminus of RNF126 interacted with ATR. Map of ATR interaction domains of RNF126. Different GST fused RNF126 fragments and FLAG-ATR were expressed in HEK293T cells. Then GST-pulldown analysis of the association between RNF126 fragments and ATR. F) Deletion of RNF126 did not affect the assembly of MRN complex. FPLC analysis of the native protein complex. Cellular extracts from RNF126 wildtype or knockout HCC1806 cells were fractionated on Superose 6 size-exclusion columns with high-salt buffer. Western blot analysis the chromatographic fractions with antibodies against the indicated proteins. Equal volumes from each fraction were analyzed. The amounts of the indicated proteins were quantified using ImageJ.

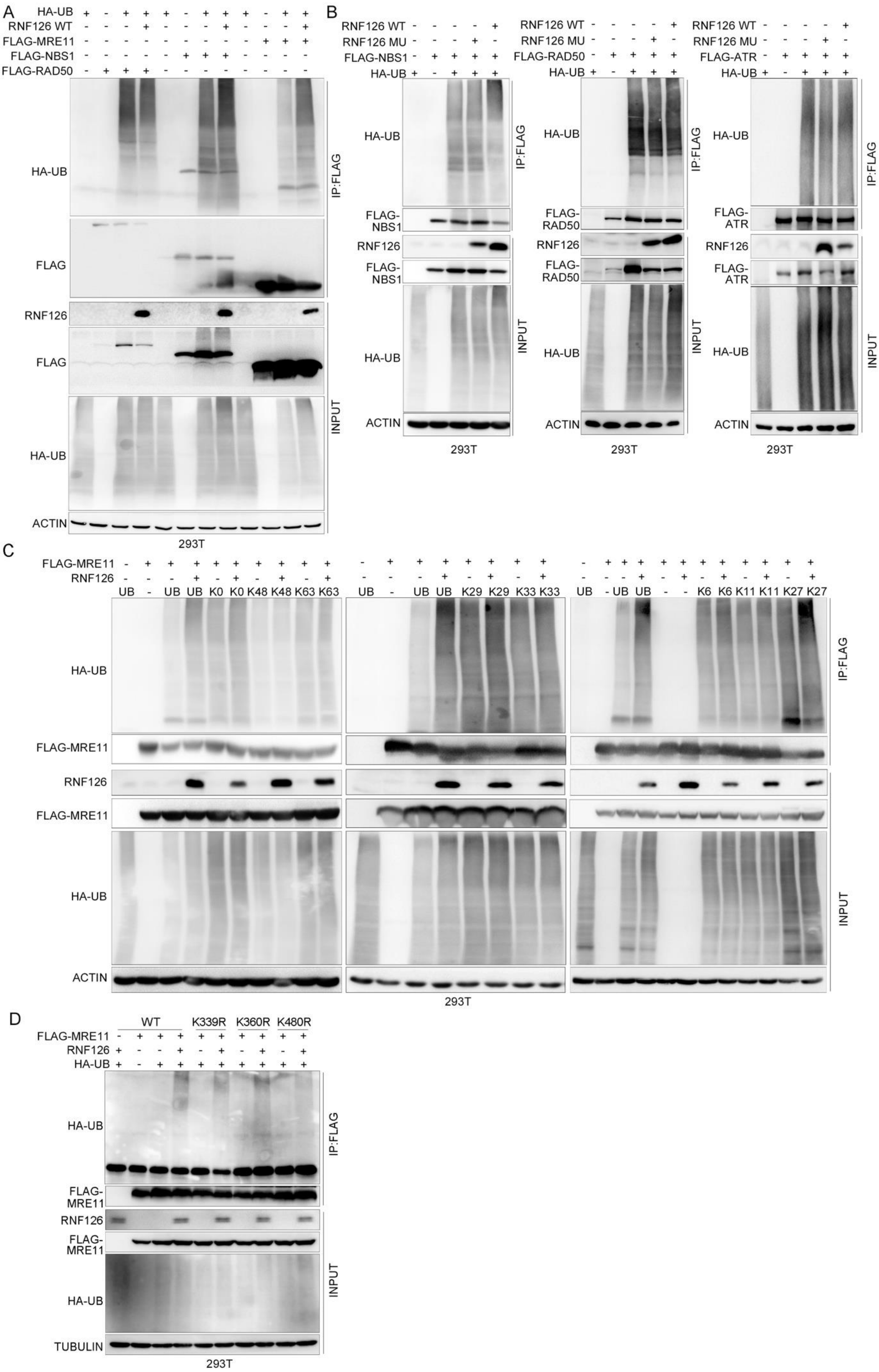
A
HA-UB
RNF126 WT
FLAG-MRE11
FLAG-NBS1
FLAG-RAD50
HA-UB
FLAG
RNF126
FLAG
HA-UB
ACTIN
IP:FLAG
INPUT
293T
B
RNF126 WT
RNF126 MU
FLAG-NBS1
HA-UB
HA-UB
FLAG-NBS1
RNF126
FLAG-NBS1
HA-UB
ACTIN
293T
FLAG-RAD50
FLAG-RAD50
RNF126
FLAG-RAD50
FLAG-ATR
FLAG-ATR
RNF126
FLAG-ATR
IP:FLAG
INPUT
C
FLAG-MRE11
RNF126
UB - UB UB K0 K0 K48 K48 K63 K63
UB - UB UB K29 K29 K33 K33
UB - UB UB - - K6 K6 K11 K11 K27 K27
HA-UB
FLAG-MRE11
RNF126
FLAG-MRE11
HA-UB
ACTIN
IP:FLAG
INPUT
293T
D
WT K339R K360R K480R
FLAG-MRE11
RNF126
HA-UB
HA-UB
FLAG-MRE11
RNF126
FLAG-MRE11
HA-UB
TUBULIN
IP:FLAG
INPUT
293T

Supplementary Figure 4. RNF126 ubiquities MRE11 at K339 and K480. A) RNF126 mainly increased the ubiquitination of MRE11. HEK293T cells expressing FLAG-RAD50/NBS1/MRE11 were cotransfected with HA-Ub and RNF126 WT. Cellular extracts were immunoprecipitated with anti-FLAG affinity gel followed by *in vivo* ubiquitination assay analysis of polyubiquitination of MRE11. B) Over-expression of RNF126 slightly influence on ubiquitination of NBS1, RAD50, or ATR. HEK293T cells expressing FLAG-NBS1/RAD50/ATR were cotransfected with HA-Ub and RNF126 WT/MU. Cellular extracts were immunoprecipitated with anti-FLAG affinity gels followed by *in vivo* ubiquitination assay analysis of polyubiquitination of MRE11. C) RNF126 mainly mediates K27-linked polyubiquitination of MRE11. HEK293T cells expressing FLAG-MRE11 were cotransfected with HA-Ub/ K0-/K48-/K63-/K27-/K29-/K33-/K6-/K11-/K27- only and RNF126 WT. Cellular extracts were immunoprecipitated with anti-FLAG followed by *in vivo* ubiquitination assay analysis of polyubiquitination of MRE11. D) RNF126 mainly promote polyubiquitination of MRE11 at K339 and K480. HEK293T cells expressing FLAG-MRE11 WT or FLAG-MRE11 K339R/K360R/K480R mutants were cotransfected with HA-Ub and RNF126 WT. Cellular extracts were immunoprecipitated with anti-FLAG followed by *in vivo* ubiquitination assay analysis of polyubiquitination of MRE11.

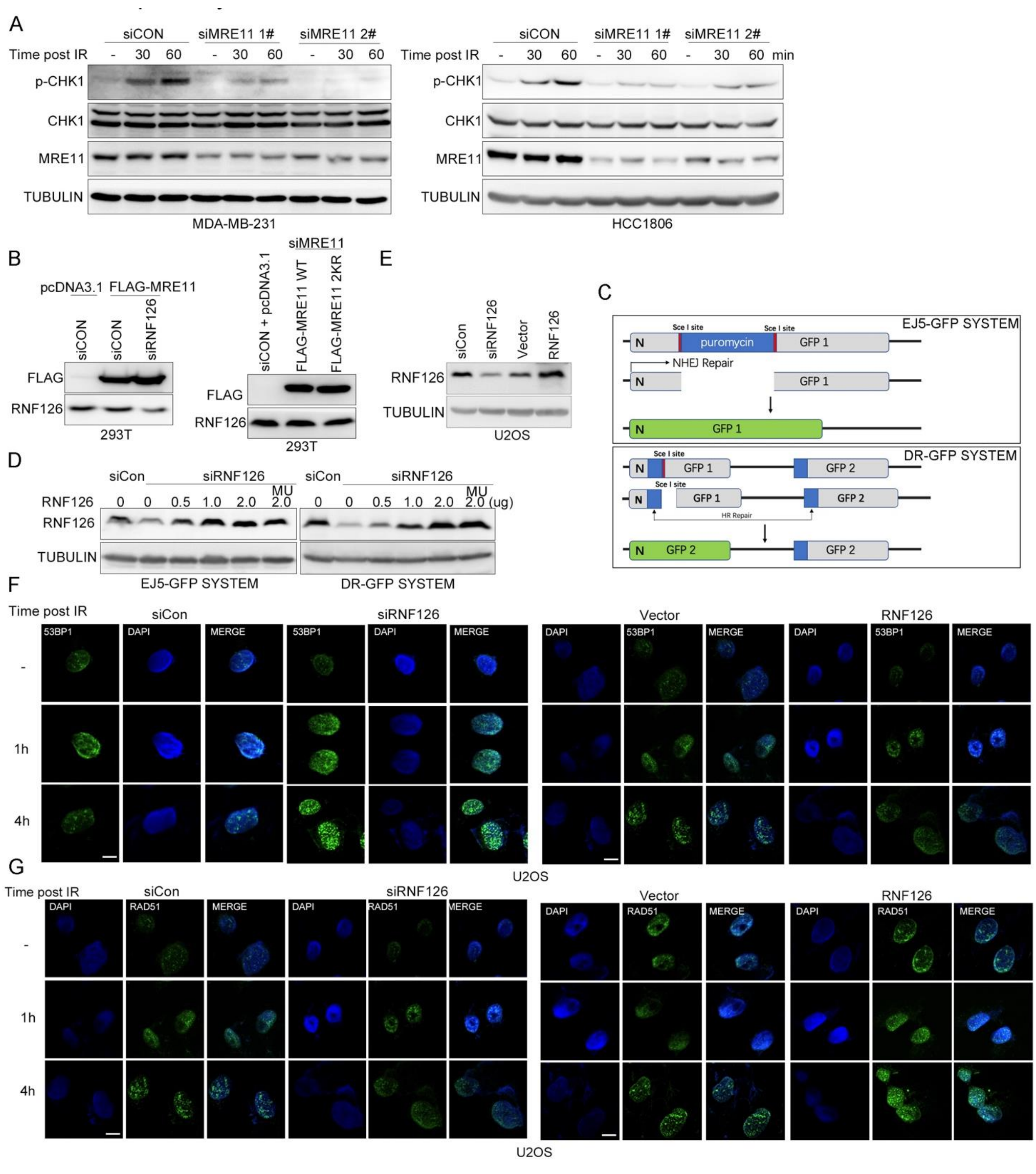

Supplementary Figure 5. RNF126 depended MRE11 ubiquitination is required for IR-induced ATR-CHK1 pathway activation. A) MRE11 knockdown-impaired CHK1 (p-Ser345) signals induced by IR. Endogenous MRE11 knockdown by siRNA in MDA-MB-231 and HCC1806 cells following treatment with IR (10 Gy). Cell lysates were harvested for western blot analysis at 0, 30 and 60 min points post IR. B) Western blotting analysis of the expression of FLAG-MRE11 and RNF126 in HEK293T cells. C) Diagram of EJ5-GFP and DR-GFP system. D) Western blotting analysis of the expression

of RNF126 in U2OS cells. E) Western blotting analysis of the expression of RNF126 in U2OS cells. F,G) Representative micrographs of 53BP1 (F) or RAD51 (G) focus formation after irradiation (10 Gy) at 0 and 4 h in the RNF126 knockdown or RNF126 overexpression U2OS cells. Scale bars, 10 μm.

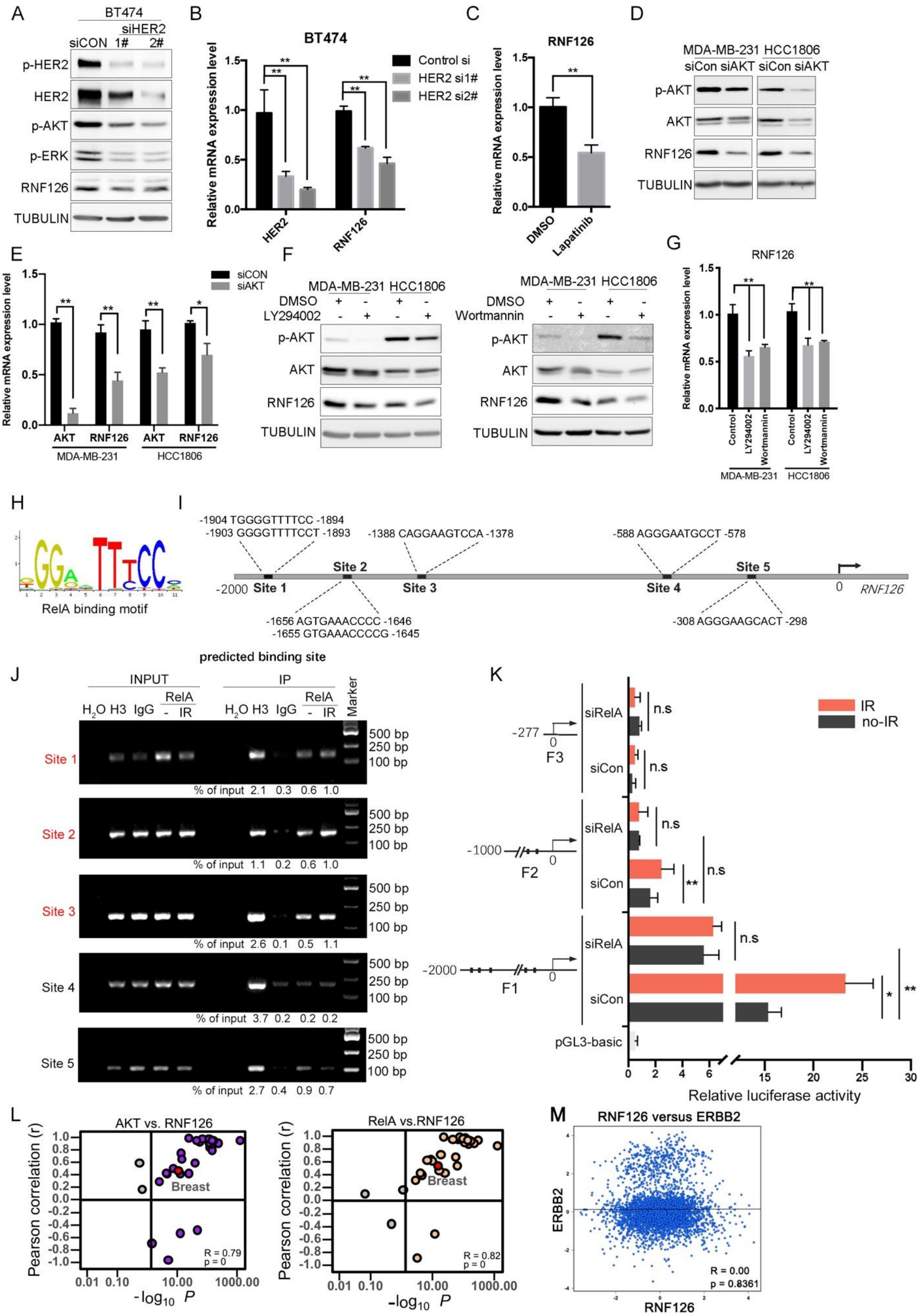

A
BT474
siCON 1# 2#
siHER2
p-HER2
HER2
p-AKT
p-ERK
RNF126
TUBULIN
B
BT474
Control si
HER2 si1#
HER2 si2#
Relative mRNA expression level
HER2
RNF126
C
RNF126
DMSO
Lapatinib
D
MDA-MB-231 HCC1806
siCon siAKT siCon siAKT
E
siCON
siAKT
AKT RNF126 AKT RNF126
MDA-MB-231 HCC1806
F
MDA-MB-231 HCC1806
DMSO
LY294002
Wortmannin
G
RNF126
Control
LY294002
Wortmannin
H
RelA binding motif
I
-1904 TGGGGTTTTCC -1894
-1903 GGGGTTTTCCT -1893
-1388 CAGGAAGTCCA -1378
-588 AGGGAATGCCT -578
Site 1
Site 2
Site 3
Site 4
Site 5
-2000
0
RNF126
-1656 AGTGAAACCCC -1646
-1655 GTGAAACCCCG -1645
-308 AGGGAAGCACT -298
predicted binding site
J
INPUT
IP
H2O H3 IgG RelA - IR
Marker
500 bp
250 bp
100 bp
% of input 2.1 0.3 0.6 1.0
% of input 1.1 0.2 0.6 1.0
% of input 2.6 0.1 0.5 1.1
% of input 3.7 0.2 0.2 0.2
% of input 2.7 0.4 0.9 0.7
K
IR
no-IR
siRelA
siCon
F3
F2
F1
pGL3-basic
n.s
Relative luciferase activity
L
AKT vs. RNF126
RelA vs.RNF126
Pearson correlation (r)
Breast
R = 0.79
p = 0
R = 0.82
p = 0
-log10 P
M
RNF126 versus ERBB2
ERBB2
RNF126
R = 0.00
p = 0.8361

Supplementary Figure 6. RNF126 expression is induced by IR activating HER2-AKT-NF-κB pathway. A) Knockdown HER2 decreased the RNF126 expression in BT474 cells. BT474 cells transfected with siCON or siHER2 and cell lysates were harvested for Western blotting analysis of p-HER2, HER2, p-AKT, p-ERK and RNF126. B) Knockdown HER2 decreased the RNF126 expression in BT474 cells. BT474 cells transfected with siCON or siHER2 and harvested for RT-qPCR analysis. C) Lapatinib decreased the RNF126 expression in BT474 cells. BT474 cells were treated with 10 μM lapatinib for 12 h and harvested for RT-qPCR analysis. D) Knockdown AKT decreased the RNF126 expression in MDA-MB-231 and HCC1806 cells. MDA-MB-231 and HCC1806 cells were transfected with siCON or siAKT and cell lysates were harvested for Western blotting analysis. E) Knockdown AKT decreased the RNF126 expression in MDA-MB-231 and HCC1806 cells. MDA-MB-231 and HCC1806 cells were transfected with siCON or siAKT and harvested for RT-qPCR analysis. F) Inhibited AKT pathway decreased the RNF126 expression in MDA-MB-231 and HCC1806 cells. MDA-MB-231 and HCC1806 cell lines were treated with LY294002 (10 μM) and Wortmannin (10 μM) and harvested for Western blotting analysis. G) Inhibited AKT pathway decreased the RNF126 expression in MDA-MB-231 and HCC1806 cells. MDA-MB-231 and HCC1806 cell lines were treated with LY294002 (10 μM) and Wortmannin (10 μM) and harvested for RT-qPCR analysis. H) The sequence logo of RelA binding motif. I) Predicted RelA binding sites within RNF126 promoter between -2000 to 0 of TSS. J) RelA bound to the RNF126 promoter region and responded to IR treatment. ChIP PCR of the RNF126 promoter using anti-RelA, anti-Histone H3 (H3, positive control) or immunoglobulin G (IgG, negative control) in HCC1806 cells treated with or without IR (10 Gy). Predicted RelA binding site1-3 within RNF126 promoter were response to radiation and were highlighted in red. K) The RNF126 promoter fragment containing prediction sites 1-3 increased promoter activity in response to IR treatment. RNF126 promoter activity was analyzed in indicated HCC1806 cells treated with or without IR (10 Gy). Error bars indicate the s.d. ($n = 3$). Statistical analysis was performed using the two-tailed, unpaired Student's t-test. * $p < 0.05$ and ** $p < 0.01$; n.s, not significant. L) RNF126 was positively correlated with RelA and AKT in normal tissues. Correlation of RNF126 with RelA and AKT in expression in normal tissues based on the data from Genotype Tissue Expression (GTEx). Note that every dot represents one tissue type. M) Correlation of RNF126 with ERBB2 (HER2) in mRNA expression in 4,421 breast cancer patients based on the data from *bc-GenExMiner v4.8*.

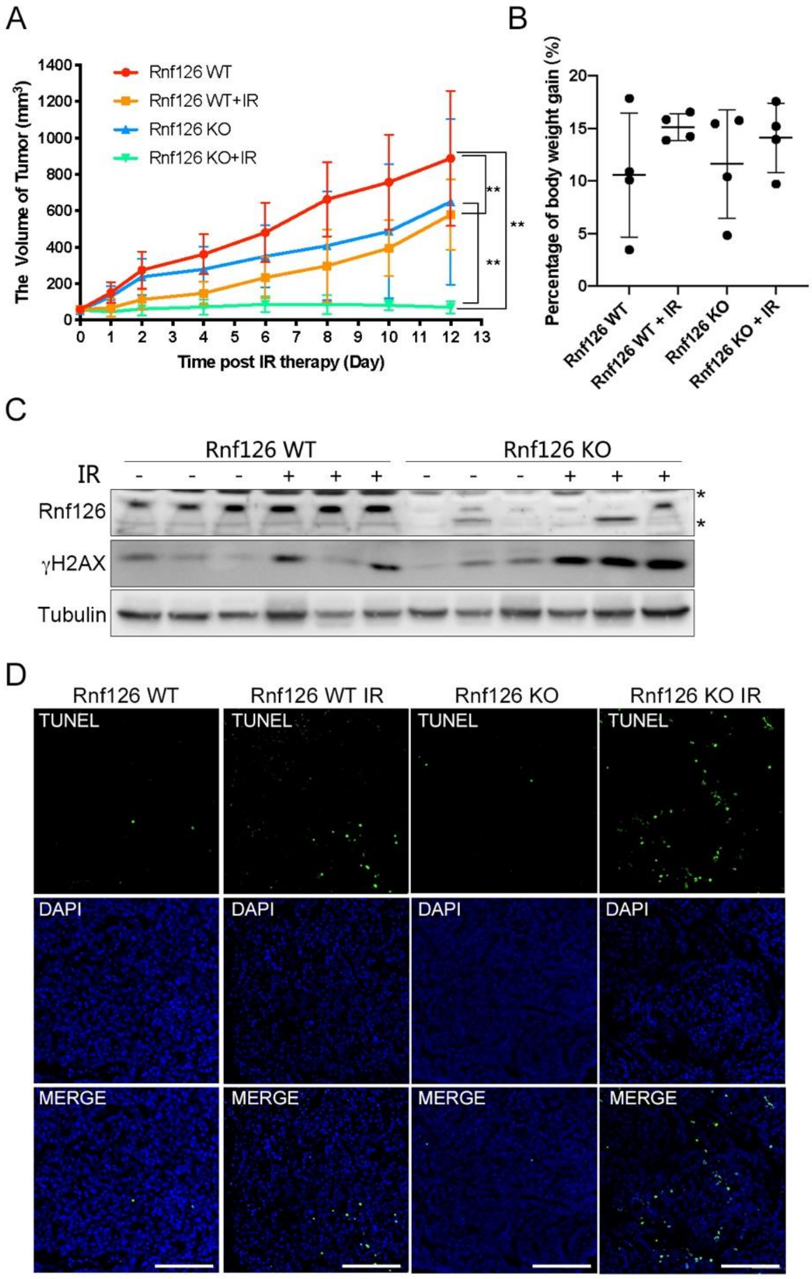

Supplementary Figure 7. PyMT-induced RNF126 WT/KO tumors in mice. A) Mice with PyMT-induced Rnf126 WT or Rnf126 KO tumors were divided into two groups, control or irradiation therapy (*n*=4 per group). Tumors were measured every two days using a vernier calliper and the volume was calculated according to the formula: $\pi/6 \times \text{length} \times \text{width}^2$. B) The proportion of body weight change from pre-treatment to sacrifice in PyMT induced breast cancer model. C) Western blotting analysis of Rnf126 expression level in indicated PyMT-induced tumor. D) Representative images of TUNEL staining of PyMT-induced tumors receiving the indicated treatment. Scale bar, 100 μm.

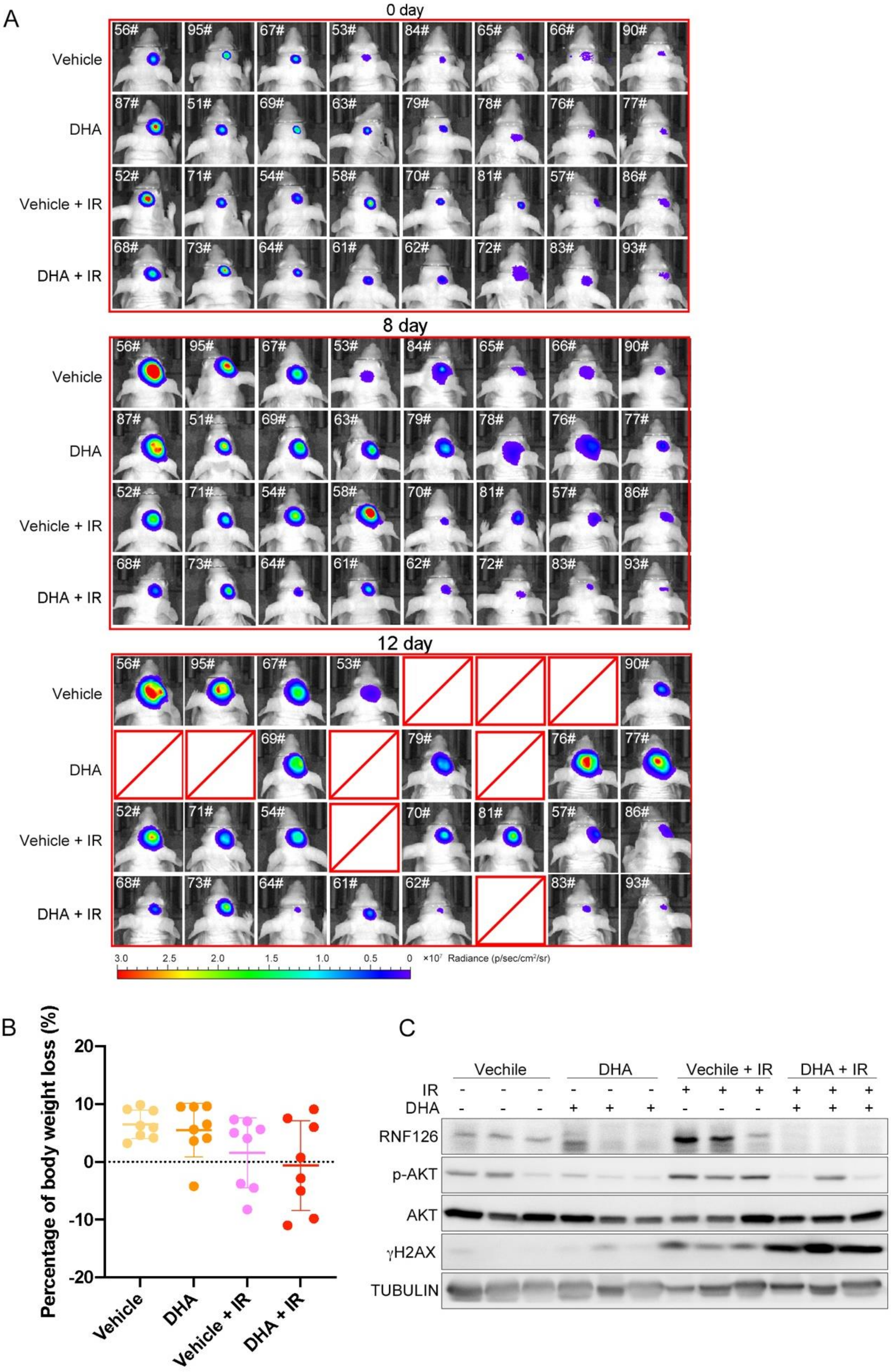

Supplementary Figure 8. The combination of DHA and irradiation improved the therapeutic benefit of irradiation therapy. A) Images of tumors in the brain imaged by IVIS at 0-, 8- and 12- day points. B) The proportion of body weight

change from pre-treatment to sacrifice in TNBC brain bearing mice model. C) Western blotting analysis the protein level of RNF126, p-AKT, AKT, γH2AX in indicated intracranial MDA-MB-231-luc tumors.

Supplementary Experimental Section

RT-qPCR: RNA was extracted using TRIzol reagent (15596-026, Invitrogen). Reverse transcription was performed using the iScript cDNA Synthesis Kit (Bio-Rad Laboratories, Hercules, CA), and RNA levels were quantified using SYBR Green Select Master Mix system (4472908, Applied Biosystems, USA) on the ABI-7900HT System (Applied Biosystems) with the expression of 18S as the internal control.

Immunofluorescence: Cells on glass coverslips were fixed with 4% para-formaldehyde and permeabilized with 0.2% Triton X-100 in PBS. Samples were blocked in 5% BSA in the presence of 0.1% Triton X-100 and stained with the appropriate primary and secondary antibodies coupled to Alexa Fluor 488, 594 (ABclonal). The primary antibodies were diluted in phosphate buffer saline with 0.1% Tween-20 (PBST) containing 5% BSA and incubated with cells overnight at 4 °C. After washing, secondary antibodies were diluted in PBST containing 5% BSA and incubated with cells at room temperature for 1 h. Then cells were washed twice and incubated with DAPI (HY-D0814, MCE, Shanghai, China) at room temperature for 5 min. After twice washing, cells were mounted with anti-fade mounting medium. Confocal images were captured on a Nikon A1MP+ Microscope with a ×63 oil objective. TUNEL staining was performed using in situ cell death detection kit (Roche). The foci in IR-treated and control cells were counted using ImageJ and FoCo.

Immunoprecipitation: Cell lysates were prepared by incubating the cells in lysis buffer (50 mM Tris-HCl, pH 8.0, 150 mM NaCl, 0.2% Nonidet P-40, 2 mM EDTA) in the presence of protease inhibitor Cocktails (MCE) for 20 min on ice. This was followed by centrifugation at 12,000 g for 15 min at 4 ℃. For immunoprecipitation, protein was incubated with control or specific antibodies (1-2 µg) for 12 hours at 4 ℃ with constant rotation; 50 µl of 50% protein A/G magnetic beads (Invitrogen) was then added and the incubation was continued for an additional 2 hours. Beads were then washed five times using the lysis buffer. Between washes, the beads were collected by magnetic stand (Invitrogen) at 4 ℃. The precipitated proteins were eluted from the beads by re-suspending the beads in 1 × SDS-PAGE loading buffer and boiling for 5 min at 98 ℃. The boiled immune complexes were subjected to SDS-PAGE followed by immunoblotting with appropriate antibodies.

Immunopurification and Silver Staining: Lysates from HCC1806 cells stably expressing FLAG-RNF126 were prepared by incubating the cells in lysis buffer containing protease inhibitor cocktail (MCE). Cell lysates were obtained from about 25×10^{7} cells, and after binding with anti-Flag M2 affinity gel (Sigma) for 2 hours as recommended by the manufacturer, the affinity gel was washed with cold lysis plus 0.1% Nonidet P-40. FLAG peptide (Sigma) was applied to elute the FLAG protein complex as described by the vendor. The elutes were collected and visualized on NuPAGE 4%-12% Bis-Tris gel (Invitrogen) followed by silver staining with silver staining kit (Pierce). The distinct protein bands were retrieved and analyzed by LC-MS/MS.

FPLC Chromatography: Cells extracts were applied to a Superose 6 size exclusion column (GE healthcare) that had been equilibrated with dithiothreitol-containing buffer. The column was eluted at a flow rate of 0.5 ml/min and fractions were collected.

Colony Formation Assay: Cells (1,000) were plated in triplicate in each well of six-well plates. Then, 16 h later, cells were treated with X-ray irradiation and cultured for 10–14 d at 37 °C to allow colony formation. Colonies were stained with crystal violet and counted.

Apoptosis: The apoptosis was analyzed with an FITC Annexin V Apoptosis Detection Kit (556,547, BD Pharmingen, California, USA) by Accuri C6 flow cytometry (BD Biosciences, California, USA). The apoptosis rate was quantified using a BD FACSAria with Cell Quest research software.

*ChIP assay：*ChIP assays were performed using the SimpleChIP® Plus Enzymatic Chromatin IP Kit (#9005, Cell Signaling Technology) according to the manufacturer’s instructions. A rabbit anti-RelA antibody (#8242, Cell Signaling Technology), a rabbit anti-Histone H3 (#4620, Cell Signaling Technology) and a normal rabbit IgG (#2729, Cell Signaling Technology) were used for immunoprecipitation. The amounts of the PCR products were quantified using ImageJ. Precipitated DNAs were subjected to PCR using the following specific primers:
site1: sense, 5′-CTGCACTTGCGGAGCGTGAAGGCT-3′ and antisense, 5′-TCTTTGCAGCAGCGCGAATCCAGT-3′;
site2: sense, 5′-TAAATGCAGGCCGGGCGCGGTGGC-3′ and antisense, 5′-GGGATTACAGGCTGGGCACCACCA-3′;
site3: sense, 5′-CAGTCTCGCTCCAATGCCTCATTT-3′ and antisense, 5′-GAGCGTGGAACTCAAGGGTGA-3′;
site4: sense, 5′-CCCGTGGAGCATCTTGGAAACAAG-3′ and antisense, 5′-GATTTTTGGACCCGCAGCAGCGCC-3′;
site5: sense, 5′-GGACAGTGGGCTGAAGTACC-3′ and antisense, 5′-CCTCCTCCCCTCCCGAAG-3′.

Dual-luciferase reporter assay: The reporter plasmids (pGL3, Promega) used contained firefly luciferase gene under the control of different RNF126 promoter fragment. Renilla luciferase was used as an internal control for normalization. HCC1806 cells were seeded into 24-well plates and transfected with indicated siRNA. 24 hours after siRNA transfection, the reporter plasmid was co-transmuted with the internal control plasmid in the presence or absence of IR (10 Gy). Luciferase activity was measured using the Dual Luciferase Reporter Assay System (E1910, Promega) according to the manufacturer's instructions. Luminescence was measured in a Luminoskan Ascent Luminometer (Thermo fisher scientific).

Mice tumor models induced by lentivirus expressing PyMT: PyMT (polyoma middle T-antigen) activates several important cellular oncogenic signaling such as PI3K/AKT and Ras/MEK/ERK through Src-mediated PyMT tyrosine phosphorylation (pY315 and pY250) to mediate carcinogenesis. Lentivirus carrying the PyMT oncogene with the Cre expression cassette was used here to induce mammary gland tumor in *Rnf126*$^{fl/fl}$ or FVB wild-type mice. Cells infected with the virus will express both PyMT and Cre, knocking out *Rnf126* in the presence of floxP sites. The PyMT with Cre expression cassette (FU-CGW) lentiviral and packing plasmids used here were described in a previous study[1,2]. The 4th mammary glands of FVB *Rnf126*$^{fl/fl}$ or FVB wild type mice were chosen for injection of the lentivirus. Following anesthetization, mice mammary ducts were exposed by removal of the nipples, and then concentrated viruses (8 µl) were injected directly into the ductal lumen of glands with a 33-gauge needle (Hamilton, Reno, NV). Trypan blue was added to trace the distribution of viral solution. The tumor was surgically removed and digested after the tumor reached 1 cm in diameter. Then, 6×10^5 PyMT induced Rnf126 WT or Rnf126 KO viable tumor cells in 75 µl PBS with Matrigel were injected into the 6-week-old female mice (FVB). After tumors grew to 70 cm^3, tumors were received 12 Gy (4 Gy, 3 times) of IR exposure for half mice in each group then tumor growth and mice weight were monitored. Four animals per group were used in each experiment. Tumors were measured every two days using a vernier calliper and the volume was calculated according to the formula: $\pi/6 \times \text{length} \times \text{width}^2$. The measurement and data processing were done with blinding. All animal experiments were performed according to the institutional ethical guidelines of animal care and were approved by Animal Resource Center at the Kunming Institute of Zoology.

Immunohistochemical staining: Paraffin-embedded clinical breast cancer specimens were obtained from the Second Xiangya Hospital of Central South University. Informed consent was obtained from all subjects (The Ethics Committee of the Second Xiangya Hospital, Central South University; Approval No. 2021-443). RNF126 antibody was used at a dilution of 1:50 and

p-RelA antibody was used at a dilution of 1:100. Detailed method was performed according to the protocol described in our previous study[3]. Staining patterns were interpreted by two pathologists with no prior knowledge of the clinicopathological parameters. Immunostained slides were evaluated under a light microscope.

Supplementary References

[1] W. Bu, L. Xin, M. Toneff, L. Li, Y. Li, *J Mammary Gland Biol Neoplasia* **2009**, *14* (4), 401.
[2] R. Liu, P. Shi, Z. Zhou, H. Zhang, W. Li, H. Zhang, C. Chen, *J Pathol* **2018**, *246* (4), 497.
[3] Y. Wu, J. Qin, F. Li, C. Yang, Z. Li, Z. Zhou, H. Zhang, Y. Li, X. Wang, R. Liu, Q. Tao, W. Chen, C. Chen, *J Biol Chem* **2019**, *294* (47), 17837.

Supplementary Table S1 Antibodies used in this work

ANTIBODIES	SOURCE	IDENTIFER	
ACTIN	Sigma-Aldrich	A5441	WB
AKT	Cell Signaling Technology	#4685	WB
Anti-phospho Histone H2A.X (Ser139)	Millipore	JBW301	WB
ATM	Cell Signaling Technology	#2873	WB
ATR	Abclonal	A7247	WB
CHK1	Abclonal	A7653	WB
Rabbit anti DDDDK-Tag pAb	Abclonal	AE004	WB
GST	Sigma-Aldrich	G7781	WB
HA	Abclonal	AE008	WB
IKKα	Cell Signaling Technology	#2682	WB
K27	Abcam	ab181537	WB
K63	Cell Signaling Technology	#5621	WB
MRE11	Abclonal	A2559	WB
NBS1	Abclonal	A0738	WB
Phospho-Akt (Ser473)	Cell Signaling Technology	#4060	WB, IHC
p-ATM（S1981）	Cell Signaling Technology	#2853	WB
p-CHK1 (Ser345)	Cell Signaling Technology	#2348	WB
p-CHK2 (T68)	Cell Signaling Technology	#2661	WB
p-ATR (Ser428)	Cell Signaling Technology	#2853S	WB
p-ATR (Ser428)	Abclonal	AP0676	IF
NFkB phospho S276	Abcam	ab194726	WB, IHC
p-RPA32 (S4/8)	Abcam	ab243866	WB
RAD50	Abclonal	A3078	WB, IP
RAD51	Abcam	ab133534	IF
RELA	Cell Signaling Technology	#8242	WB, ChIP
RNF126	Panora Biotech	Customize antibody	WB (mice)
RNF126 (C-1)	Santa cruz	sc-376005	IHC,WB,IP
RPA32	Abcam	ab76420	WB
RPA70	Cell Signaling Technology	#2198	IF
TP53BP1	Abclonal	A5757	IF
TUBULIN	Sigma-Aldrich	F7425	WB

Supplementary Table S2 Plasmids used in this paper

RECOMBINANT DNA	SOURCE
pBabe-RNF126	this study
pBabe-RNF126 C229/232A	this study
PCDH-RNF126	this study
PCDH-RNF126 (3# sgRNA against, atggccgaggcgtcgccgcatcccgga agatactt)	this study
PCDH-RNF126 C229/232A (3# sgRNA against, atggccgaggcgtcgccgcatcccgga agatactt)	this study
pEBG-GST-RNF126	this study
pEBG-GST-RNF126 F1 (1-100)	this study
pEBG-GST-RNF126 F2 (101-200)	this study
pEBG-GST-RNF126 F3 (201-311)	this study
PCDH-GFP-RNF126	this study
pcDNA3.1-flag-NBS1	Gift from Dr. Lei Shi (Tianjin Medical University)
pcDNA3.1-flag-RAD50	Gift from Dr. Lei Shi (Tianjin Medical University)
pcDNA3.1-flag-RAD50 F1 (1-572)	this study
pcDNA3.1-flag-RAD50 F2 (573-829)	this study
pcDNA3.1-flag-RAD50 F3 (830-1312)	this study
pcDNA3.1-flag-MRE11	Gift from Dr. Lei Shi (Tianjin Medical University)
pcDNA3.1-flag-MRE11 K339R	this study
pcDNA3.1-flag-MRE11 K360R	this study
pcDNA3.1-flag-MRE11 K480R	this study
pcDNA3.1-flag-MRE11 K339R/K480R	this study
HA-UB (HA-ubiquitin WT)	this study
K0 (HA-ubiquitin, all lysine residues were mutated)	this study
K48 (HA-ubiquitin K48)	this study
K63 (HA-ubiquitin K63)	this study
K29 (HA-ubiquitin K29)	this study
K33 (HA-ubiquitin K33)	this study
K6 (HA-ubiquitin K6)	this study
K11 (HA-ubiquitin K11)	this study
K27 (HA-ubiquitin K27)	this study
K27R (HA-ubiquitin mutant K27R)	this study

K29R (HA-ubiquitin mutant K29R)	this study
K63R (HA-ubiquitin mutant K63R)	this study
pGL3-basic	Promega
pGL3-RNF126 promoter F1	this study
pGL3-RNF126 promoter F2	this study
pGL3-RNF126 promoter F3	this study
